# Clinical practice guideline for aniridia

**DOI:** 10.1007/s10384-025-01296-y

**Published:** 2026-03-28

**Authors:** Research on rare and intractable diseases, Health, Labour and Welfare Sciences Research Grants Clinical Practice Guideline Development Committee for Aniridia of the “Research group on establishing standardized diagnosis and treatment of Intractable corneal diseases”

**Affiliations:** Osaka, Japan

## Preface

Aniridia is an intractable eye disease characterized by varying degrees of iris hypoplasia complicated by keratopathy, cataract, glaucoma, macular hypoplasia, and nystagmus. The responsible gene is *PAX6*, known as the master gene in ocular development; aniridia occurs when the amount of functional gene is halved (haploinsufficiency) because of the loss of function of one allele.

The Act on Medical Care for Patients with Intractable Diseases (Intractable/Rare Disease Act) in Japan designates aniridia as an intractable disease, and the diagnostic criteria and severity classification of aniridia were defined by a research group charged with establishing a standardized diagnosis and treatment of intractable corneal diseases. We have now developed the clinical practice guidelines using the Medical Information Network Distribution Service (Minds) method to ensure that high-quality medical care can be provided to patients with aniridia. Minds is part of the Promotion Project for Evidence-Based Medicine, operated by the Japan Council for Quality Health Care. It was commissioned by the Ministry of Health, Labour and Welfare in Japan.

Minds defines clinical practice guidelines as Documents that, based on systematic reviews of the evidence, their overall assessment, and the balance between benefits and harms, can provide healthcare professionals in medical practice of high clinical importance with the optimum recommendations to support decision-making for the treatment of patients.

Therefore, instead of collecting expert opinions, we conducted systematic reviews to collect evidence, and then evaluated and summarized all available evidence to ensure a comprehensive and objective guidelines’ development process. To ensure an unbiased judgment process in the development of clinical practice guidelines, it is recommended that tasks be divided and carried out by three groups: clinical practice guidelines supervisory committee, clinical practice guidelines development group, and systematic review team. The clinical practice guidelines supervisory committee will supervise the overall development. The clinical practice guideline development group will be responsible for defining the scope and identifying the key clinical questions (CQs). The systematic review team will conduct systematic reviews based on the identified CQs and evaluate the overall body of evidence. The organizational structure and roles in the development of these clinical practice guidelines are detailed in Table 1.

**Table 1:** Guideline development organizations

(1) Main body for the development of the clinical practice guideline	Name of academic society/study group	Research program on rare and intractable diseases, Health, Labour and Welfare Sciences Research Grants; the “Research group on establishing standardized diagnosis and treatment of intractable corneal diseases”		
	Name of related/collaborating academic society	Japanese Ophthalmological Society		
	Name of related/collaborating academic society	Japan Cornea Society		
	Name of related/collaborating academic society	Japanese Association of Pediatric Ophthalmology		
(2) Clinical practice guideline supervisory committee	Representative	Name	Affiliation/specialty	Role
	〇	Kohji Nishida	Department of Ophthalmology, The University of Osaka Graduate School of Medicine/Ophthalmology	Supervision of guideline development
		Akira Murakami	Department of Ophthalmology, Juntendo University Graduate School of Medicine/Ophthalmology	Instruction on guideline development
		Noriyuki Azuma	Department of Ophthalmology and Laboratory for Visual Science, National Center for Child Health and Development/Ophthalmology	Instruction on guideline development
		Jun Shimazaki	Department of Ophthalmology, Tokyo Dental College, Ichikawa General Hospital/Ophthalmology	Instruction on guideline development
		Kazunori Miyata	Miyata Eye Hospital, Medical Corporation Meiwakai/Ophthalmology	Instruction on guideline development
		Masakazu Yamada	Department of Ophthalmology, Kyorin University School of Medicine/Ophthalmology	Instruction on guideline development
		Chie Sotozono	Department of Ophthalmology, Kyoto Prefectural University of Medicine/Ophthalmology	Instruction on guideline development
		Atsushi Shiraishi	Department of Ophthalmology, Ehime University School of Medicine/Ophthalmology	Instruction on guideline development
		Shigeto Shimmura	Department of Ophthalmology, Keio University School of Medicine/Ophthalmology	Instruction on guideline development
		Tomohiko Usui	Department of Ophthalmology, The University of Tokyo Hospital/Ophthalmology	Instruction on guideline development
		Yoshinori Oie	Department of Ophthalmology, The University of Osaka Graduate School of Medicine/Ophthalmology	Instruction on guideline development
(3) Secretariat for clinical practice guideline development	Representative	Name	Affiliation/specialty	Role
	〇	Yoshinori Oie	Department of Ophthalmology, The University of Osaka Graduate School of Medicine/Ophthalmology	Review of public comments, disclosure of guideline
		Nozomi Nishida	Department of Ophthalmology, The University of Osaka Graduate School of Medicine/Ophthalmology	Review of public comments, disclosure of guideline
(4) Clinical practice guideline development group	Representative	Name	Affiliation/specialty	Role
	〇	Kohji Nishida	Department of Ophthalmology, The University of Osaka Graduate School of Medicine/Ophthalmology	Guideline development
		Akira Murakami	Department of Ophthalmology, Juntendo University Graduate School of Medicine/Ophthalmology	Guideline development
		Noriyuki Azuma	Department of Ophthalmology and Laboratory for Visual Science, National Center for Child Health and Development/Ophthalmology	Guideline development
		Jun Shimazaki	Department of Ophthalmology, Tokyo Dental College, Ichikawa General Hospital/Ophthalmology	Guideline development
		Atsushi Shiraishi	Department of Ophthalmology, Ehime University School of Medicine/Ophthalmology	Guideline development
		Tomohiko Usui	Department of Ophthalmology, University of Tokyo Hospital/Ophthalmology	Guideline development
		Yoshinori Oie	Department of Ophthalmology, The University of Osaka Graduate School of Medicine/Ophthalmology	Guideline development
(5) Systematic review team	Representative	Name	Affiliation/specialty	Role
	〇	Tomomi Yamada	Department of Medical Innovation, The University of Osaka Hospital/Biostatistics	Supervision of systematic review
		Akira Matsuda	Department of Ophthalmology, Juntendo University Graduate School of Medicine/Ophthalmology	Systematic review
		Kanji Hori	Department of Ophthalmology, Juntendo University Graduate School of Medicine/Ophthalmology	Systematic review
		Toshimitsu Kasuga	Department of Ophthalmology, Juntendo University Graduate School of Medicine/Ophthalmology	Systematic review
		Takefumi Yamaguchi	Department of Ophthalmology, Tokyo Dental College, Ichikawa General Hospital/Ophthalmology	Systematic review
		Daisuke Tomida	Department of Ophthalmology, Tokyo Dental College, Ichikawa General Hospital/Ophthalmology	Systematic review
		Yuko Hara	Department of Ophthalmology, Ehime University School of Medicine/Ophthalmology	Systematic review
		Yasuhito Hayashi	Department of Ophthalmology, Ehime University School of Medicine/Ophthalmology	Systematic review
		Takashi Miyai	Department of Ophthalmology, The University of Tokyo Hospital/Ophthalmology	Systematic review
		Junko Yoshida	Department of Ophthalmology, The University of Tokyo Hospital/Ophthalmology	Systematic review
		Takahiro Minami	Department of Ophthalmology, The University of Tokyo Hospital/Ophthalmology	Systematic review
		Hitoha Ishii	Department of Ophthalmology, The University of Tokyo Hospital/Ophthalmology	Systematic review
		Yumi Hashimoto	Department of Ophthalmology, The University of Tokyo Hospital/Ophthalmology	Systematic review
		Mayumi Sainohira	Department of Ophthalmology, Kagoshima University Graduate School of Medical and Dental Sciences/Ophthalmology	Systematic review
		Shimpei Komoto	Department of Ophthalmology, Yodogawa Christian Hospital/Ophthalmology	Systematic review
		Motokazu Tsujikawa	Department of Biomedical Informatics, The University of Osaka Graduate School of Medicine, Division of Health Sciences/Ophthalmology	Systematic review
		Ryo Kawasaki	Department of Vision Informatics, The University of Osaka Graduate School of Medicine/Ophthalmology	Systematic review
		Kenji Matsushita	Department of Ophthalmology, The University of Osaka Graduate School of Medicine/Ophthalmology	Systematic review
		Yoshinori Oie	Department of Ophthalmology, The University of Osaka Graduate School of Medicine/Ophthalmology	Systematic review
		Sanae Asonuma	Department of Ophthalmology, The University of Osaka Graduate School of Medicine/Ophthalmology	Systematic review
		Hiroyuki Kurakami	Department of Medical Innovation, The University of Osaka Hospital/Statistics	Systematic review
(6) External review committee	Representative	Name	Affiliation/specialty	Role
		Yuichi Hori	Department of Ophthalmology, Toho University Omori Medical Center/Ophthalmology	Review of guideline
		Toshiyuki Ojima	Department of Community Health and Preventive Medicine, Hamamatsu University School of Medicine/Public health and epidemiology	Review of guideline

In these clinical practice guidelines, we summarize the evidence for 6 clinical questions (CQs) and 3 background questions (BQs), and make recommendations on the CQs (Table 2). Because it is a rare disease randomized controlled trials and other high-evidence studies have not been conducted on aniridia; thus, strong recommendations could not be made for many of the CQs. However, as specified as a goal of Minds, we hope that these clinical practice guidelines will help patients and healthcare professionals to discuss information on treatment options that are considered scientifically appropriate, and help them to agree on and select the best approach while considering the patients’ wishes and beliefs, the healthcare professionals’ code of ethics, and social constraints.

**Table 2:** Guideline summary

CQ no.	CQ	Summary and recommendation	Level of recommendation
1	Is keratoplasty recommended for corneal stromal opacity in aniridia?	Weak recommendation not to perform keratoplasty for corneal stromal opacity in aniridia. The improvement in visual function following a corneal transplant is limited because of the comorbidities of aniridia. In addition, the long-term prognosis for visual acuity is often poor due to glaucoma and graft dysfunction over time.	Weak recommendation “not to implement”
2	Is surgical treatment recommended for limbal stem cell deficiency in aniridia?	Weak recommendation to implement surgical treatment for limbal stem cell deficiency in aniridia. Specifically, some successful ocular surface reconstruction can be expected by performing allogeneic limbal transplantation or cultivated oral mucosal epithelial transplantation. In addition, when corneal stromal opacity is present, a combination with a corneal transplant is often useful for improving visual acuity.	Weak recommendation “to implement”
3	Is surgical treatment recommended for cataract in aniridia?	Although some patients with aniridia may experience an improvement in visual acuity following cataract surgery in aniridia, surgery is difficult because of the fragility associated with the capsule and the Zonule of Zinn. In addition, there is a high risk of possible worsening of postoperative glaucoma, anterior fibrosis syndrome, and bullous keratopathy. Therefore, it is recommended that one considers the risks associated with surgery and provides sufficient information to the patient in advance.	Weak recommendation “to implement”
4	What treatment options are appropriate for high intraocular pressure and glaucoma in aniridia?	To lower intraocular pressure, the following approaches should be used: (1) intraocular pressure-lowering therapy with drugs such as eye drops and oral drugs, (2) angle surgery (goniotomy or trabeculotomy), (3) filtration surgery (mainly trabeculectomy), (4) glaucoma implant surgery, and (5) ciliary body coagulation. When selecting a treatment approach, first consider drug therapy, such as eye drops and oral drugs, and thereafter pay attention to adverse effects; if treatment is ineffective, consider angle surgery. If angle surgery is difficult or unsuccessful, trabeculectomy or glaucoma implant surgery should be considered; when deciding which treatment to implement, it is recommended that one considers factors such as the condition of the affected eye, the surgeon’s level of experience, and whether or not the facility has been certified for glaucoma implant surgery. If these treatments are unsuccessful, ciliary body coagulation may be performed, but only if the benefits outweigh the risk of complications of poor visual prognosis, such as phthisis bulbi.	Strong recommendation “to implement”
5	What is the recommended form of low vision rehabilitation in aniridia?	The basis for low vision rehabilitation is refraction correction for ametropia, which aims to improve visual function in aniridia. It is also recommended that one uses additional low vision devices, such as magnifiers, tinted lenses, low vision glasses, closed circuit television, and Iris Lenses.	Strong recommendation “to implement”
6	What is the recommended treatment for photophobia in aniridia?	Tinted lenses and Iris Lenses are recommended as treatments for photophobia in aniridia.	Strong recommendation “to implement”

Research on rare and intractable diseases, Health, Labour and Welfare Sciences Research Grants.

The research group of “Intractable corneal diseases on establishing standardized diagnosis and treatment”.

Kohji Nishida, Principal Investigator.

## List of authors

**Table:** 

*Chairperson*	
Kohji Nishida	Department of Ophthalmology, The University of Osaka Graduate School of Medicine
*Committee members (in alphabetical order)*	
Akira Matsuda	Department of Ophthalmology, Juntendo University Graduate School of Medicine
Akira Murakami	Department of Ophthalmology, Juntendo University Graduate School of Medicine
Atsushi Shiraishi	Department of Ophthalmology, Ehime University School of Medicine
Daisuke Tomida	Department of Ophthalmology, Tokyo Dental College, Ichikawa General Hospital
Hiroyuki Kurakami	Department of Medical Innovation, The University of Osaka Hospital
Hitoha Ishii	Department of Ophthalmology, The University of Tokyo Hospital
Jun Shimazaki	Department of Ophthalmology, Tokyo Dental College, Ichikawa General Hospital
Junko Yoshida	Department of Ophthalmology, The University of Tokyo Hospital
Kanji Hori	Department of Ophthalmology, Juntendo University Graduate School of Medicine
Kenji Matsushita	Department of Ophthalmology, The University of Osaka Graduate School of Medicine
Mayumi Sainohira	Department of Ophthalmology, Kagoshima University Graduate School of Medical and Dental Sciences
Motokazu Tsujikawa	Department of Biomedical Informatics, The University of Osaka Graduate School of Medicine, Division of Health Sciences
Noriyuki Azuma	Department of Ophthalmology and Laboratory for Visual Science, National Center for Child Health and Development
Ryo Kawasaki	Department of Vision Informatics, The University of Osaka Graduate School of Medicine
Sanae Asonuma	Department of Ophthalmology, The University of Osaka Graduate School of Medicine
Shimpei Komoto	Department of Ophthalmology, Yodogawa Christian Hospital
Takahiro Minami	Department of Ophthalmology, The University of Tokyo Hospital
Takashi Miyai	Department of Ophthalmology, The University of Tokyo Hospital
Takefumi Yamaguchi	Department of Ophthalmology, Tokyo Dental College, Ichikawa General Hospital
Tomohiko Usui	Department of Ophthalmology, The University of Tokyo Hospital
Tomomi Yamada	Department of Medical Innovation, The University of Osaka Hospital
Toshimitsu Kasuga	Department of Ophthalmology, Juntendo University Graduate School of Medicine
Yasuhito Hayashi	Department of Ophthalmology, Ehime University School of Medicine
Yoshinori Oie	Department of Ophthalmology, The University of Osaka Graduate School of Medicine
Yuko Hara	Department of Ophthalmology, Ehime University School of Medicine
Yumi Hashimoto	Department of Ophthalmology, The University of Tokyo Hospital
*External review committee members*	
Yuichi Hori	Department of Ophthalmology, Toho University Omori Medical Center
Toshiyuki Ojima	Department of Community Health and Preventive Medicine, Hamamatsu University School of Medicine
*Approved by Japanese Ophthalmological Society*	
Japan Cornea Society	
Japanese Association of Pediatric Ophthalmology	
*Collaborators*	
Noriaki Akai	Life Sciences Library, The University of Osaka
Nozomi Nishida	Department of Ophthalmology, The University of Osaka Graduate School of Medicine

## Medical diagram


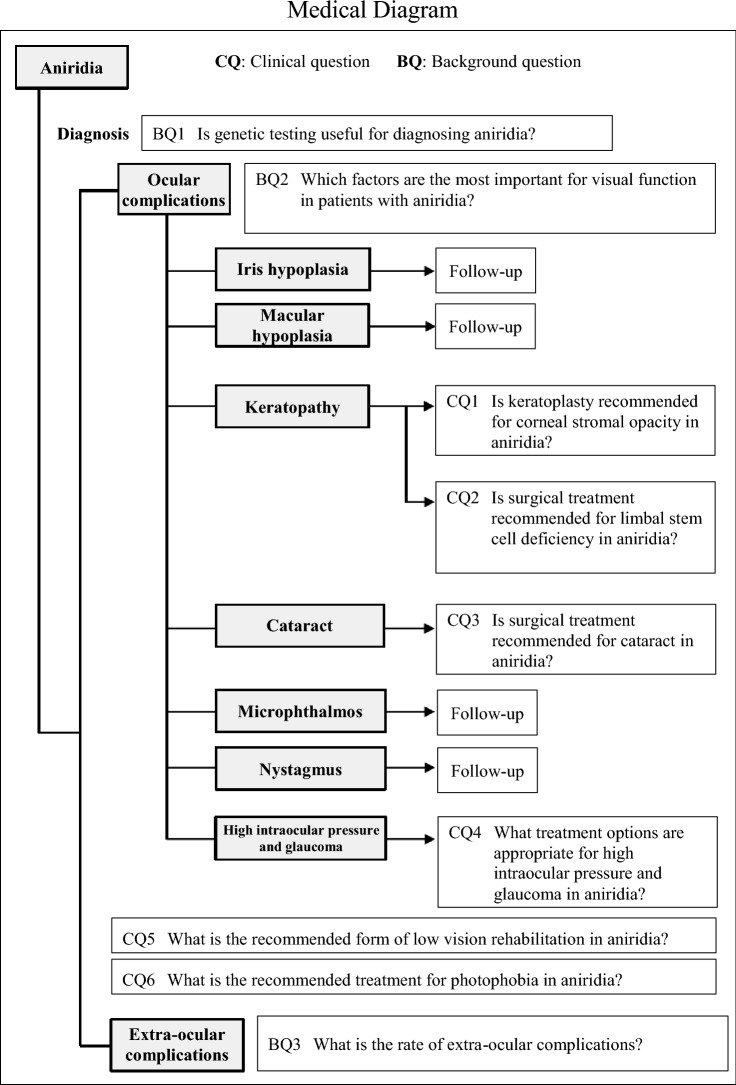


## Explanations of important terminology

**Table:** 

Term	Descriptions
Macular hypoplasia	This refers to congenitally insufficient formation of the macula of the retina. This condition is characterized by the disappearance of physiological depressions, aberrant macular vessels, and disappearance of the macular reflex on fundus examination.
Corneal stromal opacity	Corneal stromal opacity is a condition in which 1 of the 3 layers of the cornea (which consists of the epithelium, stroma, and endothelium) is opaque.
Limbal stem cell deficiency	Limbal epithelial stem cells are known to exist at the base of the limbus, which is the boundary region between the cornea and the conjunctiva. Limbal stem cell deficiency is a condition in which these cells disappear and the conjunctival epithelium, which is opaque and contains blood vessels, invades the cornea, resulting in decreased visual acuity.
Nystagmus	A condition in which both eyes show a rhythmic, involuntary oscillation.
Photophobia	Light sensitivity
Microphthalmos	This refers to a condition in which the eyeball is congenitally small and is less than two-thirds of the normal eyeball volume. The axial length is usually less than 0.87 of the normal length for age.
Low vision care	Rehabilitation for visually impaired adults and children

## Details of recommendations and explanations

### Overview

These guidelines were devised in accordance with the “Minds Manual for Guideline Development 2017.” Important clinical issues were determined by reviews conducted by the guidelines’ development committee. Issues that could be presented as recommendations were taken up in the form of clinical questions (CQs), and a systematic review (SR) was conducted for each CQ. Based on the results of each SR, a recommendation was prepared. Important clinical issues for which recommendations were difficult to make are listed as background questions (BQs), and the results were summarized for the SR performed for each BQ outcome.

**Table:** 

**Clinical question**	
A CQ is based on an important clinical issue addressed in the clinical practice guideline, developed by extracting the components (patient/population, intervention, comparison and outcomes) of each relevant question to be answered in the guideline. A CQ presents the extracted components as a question. In these guidelines, important clinical issues were divided into the 3 items diagnosis, treatment, and ocular complications to form 6 CQs.	

**Table:** 

**Presentation of recommendations**	
Based on the SR results for each CQ, recommendations were decided following deliberation by the guideline development group taking into account the “strength of evidence,” “balance between benefits and harms,” “diversity of patient values and intentions,” and “economic perspective” related to outcomes. We summarize the limited evidence and present the best policy as the recommendation even for issues for which it was difficult to make evidence-based recommendations because of the nature of rare diseases.	

**Table:** 

**Strength of recommendations**	
The strength of each recommendation was determined by the guidelines’ development group according to the method defined in the Scope section and is presented in the following 4 categories according to the direction and strength of the recommendation. Strong recommendation implementation advised Weak recommendation implementation advised Weak recommendation implementation not advised Strong recommendation implementation not advised	

**Table:** 

**Strength of evidence for the clinical questions**	
The evidence for each CQ was summarized by integrating the strength of evidence (total evidence) evaluated for each outcome. The definitions of each level of strength of evidence, A to D, are shown below. In principle, the strength of evidence was judged to be D (very weak) or C (weak) if only case reports or case series were available. A (strong):Strong confidence in the estimated effect. B (moderate):Moderate confidence in the estimated effect. C (weak):Limited confidence in the estimated effect. D (very weak):Almost no confidence in the estimated effect.	

**Table:** 

**Development process of the recommendations**	
A background is provided for the recommendations for the CQs.	

**Table:** 

**Summary of SR report**	
A commentary on how the overall strength of the evidence was determined is provided with the results of the qualitative SR.	

**Table:** 

**References**	
A list of references is provided for each SR.	

## Chapter 1

### Scope

#### I. Clinical characteristics

Aniridia is a disease caused by haploinsufficiency of the *PAX6* gene, the master gene of ocular development, due to a loss-of-function mutation in one allele [1]. The *PAX6* gene is expressed in various tissues of the eyeball during development; therefore, a diverse range of ocular complications can occur. In addition to iris hypoplasia with varying degrees of severity, keratopathy, cataract, glaucoma, macular hypoplasia, and nystagmus syndrome may also occur [2–4]. Background question (BQ) 2 summarizes the ocular complications considered to be important factors in determining visual function. In addition, extra-ocular complications such as callosal agenesis, epilepsy, higher brain dysfunction, anosmia, diabetes, and Wilms’ tumor are also known to occur [5, 6]. The incidences of these extra-ocular complications are summarized in BQ3.

#### II. Epidemiological characteristics

Aniridia is a rare disease with an estimated prevalence ranging from 1/64,000 to 1/96,000 [7, 8]. There is no difference in incidence between men and women. It is a hereditary disease with an autosomal dominant inheritance pattern. Approximately two-thirds of cases are familial, and the remaining (one-third) are sporadic.

#### III. Flow of medical care and treatment

1. Diagnosis and severity

The Research group on establishing standardized diagnosis and treatment of intractable corneal diseases has established the following diagnostic and severity grading criteria for aniridia [9]. The usefulness of genetic testing is summarized in BQ1.

1. Diagnostic criteria

A. Symptoms

1. Bilateral visual impairment (Note 1)
2. Photophobia (Note 2)

B. Test findings

1. Slit-lamp examination shows iris hypoplasia with varying degrees of severity, ranging from partial iris atrophy to complete iris deficiency (Note 3).
2. Hypoplasia of the macula is observed on fundus examination and optical coherence tomography (OCT) (Note 4).
3. Slit-lamp examination reveals corneal lesions such as limbal stem cell deficiency and corneal opacity (Note 5).
4. Slit-lamp examination findings indicate a cataract (Note 6).
5. Ultrasonography, magnetic resonance imaging (MRI), and computed tomography show microphthalmia.
6. Nystagmus is observed.
7. Glaucoma is shown by the intraocular pressure test (Note 7).

C. Differential diagnosis

1. Iris atrophy due to past infection with the herpesvirus family
2. Iris defect due to trauma or intraocular surgery
3. Iris coloboma associated with closure of the optic fissure
4. Rieger’s anomaly
5. Iridocorneal endothelial syndrome

D. Extra-ocular complications

- Abnormalities associated with the *PAX6* gene mutation (Note 8)

E. Genetic tests

- A pathogenic gene mutation or deletion of the 11p13 region of the *PAX6* gene is identified.

F. Other findings

- Other family members are affected (Note 9).

- <Diagnostic category>

- Definite: Any item under A and both B1 and E are observed, and all items under C are excluded.
- Probable: (1) Any item under A and both B1 and F are observed, and all items under C are excluded.
- (2) Any item under A and both B1 and B2 are observed, and all items under C are excluded.
- (3) Any item under A and both B1 and B3 are observed, and all items under C are excluded.
- Possible: Any item under A and B1 are observed, but items under C cannot be completely excluded.

- Note 1. Visual impairment is caused by ocular complications such as macular hypoplasia, cataract, glaucoma, and limbal stem cell deficiency.
- Note 2. Photophobia can be an accompanying symptom, depending on the degree of the iris defect.
- Note 3. 60–90% are bilateral.
- Note 4. The macula pigment, foveal depression, and foveal avascular area of the macula become obscured.
- Note 5. Depending on the disease stage, corneal lesions of varying degrees can occur, ranging from hypoplasia of the palisades of Vogt to invasion of conjunctival tissue with blood vessels and keratinization of the epithelium.
- Note 6. This complication occurs in about 80% of cases.
- Note 7. This complication occurs in 50–75% of cases due to dysplasia of the angle.
- Note 8. The *PAX6* gene is expressed not only in ocular tissues but also in the central nervous system, islands of Langerhans (in the pancreas), and olfactory epithelium. Hypoplasia of these tissues causes callosal agenesis, epilepsy, higher brain dysfunction, anosmia, and glucose intolerance. Thus, *PAX6* gene mutation may be accompanied by various extra-ocular complications.
- Note 9. Familial (autosomal dominant inheritance) aniridia accounts for two-thirds of cases, and the remaining are sporadic.

(2) Severity classification

- Grade I: One eye is affected, the fellow eye (the other eye) is healthy.
- Grade II: Both eyes are affected, the corrected visual acuity of the better eye is ≥0.3.
- Grade III: Both eyes are affected, the corrected visual acuity of the better eye is ≥0.1 and <0.3.
- Grade IV: Both eyes are affected, and corrected visual acuity of the better eye is <0.1.

- Note 1: Healthy refers to a condition in which the corrected visual acuity is ≥1.0, no visual field abnormality is observed, and no organic abnormality is observed in the eye.
- Note 2: In grades I to III, whenever secondary glaucoma is accompanied by a narrowing of the visual field of the better eye, the severity classification is moved up by one level.
- Note 3: Narrowed vision indicates that the residual visual field at the center is within 20 degrees as assessed by the Goldmann perimeter I/4 optotype.
- Note 4: If visual acuity cannot be measured in patients, e.g., infants, the severity classification should be determined comprehensively from ophthalmological findings, etc.

2. Treatment
  Although various ocular complications are observed in aniridia, follow-up is required for iris hypoplasia, macular hypoplasia, microphthalmia, and nystagmus because, in principle, there is no treatment for these conditions (refer to the Medical Diagram). The ocular complications of keratopathy (2 types: corneal stromal opacity and limbal stem cell deficiency), cataract, high intraocular pressure, and glaucoma may be treated via keratoplasty, cataract surgery, and glaucoma eye drops or surgery, respectively [10–12]. Specific details are described under clinical questions (CQs) 1 to 4. Refer to CQ5 for low vision care and CQ6 for the treatment of photophobia, which is a frequent patient complaint.

#### IV. Information described in this clinical practice guideline

1. Title

Clinical practice guideline for aniridia

2. Purpose

These clinical practice guidelines aim to improve the following outcomes:

- Diagnosis of aniridia
- Visual function in aniridia
- Keratopathy as an ocular complication
- Cataract as an ocular complication
- Glaucoma as an ocular complication
- Diagnosis of extra-ocular complications

3. Topic

Diagnosis of aniridia and clinical management of ocular complications

4. Expected users and facilities, and medical sites where the guideline may be applicable

Physicians at ophthalmology departments of university hospitals and regional core hospitals, practitioners at eye clinics, and patients

5. Relationship with existing guidelines

There are no existing clinical practice guidelines on aniridia in Japan.

6. Important clinical issues

(1) Genetic tests for aniridia

Genetic testing for pathogenic mutations in the *PAX6* gene or deletion of the 11p13 region has been performed to diagnose aniridia. However, its usefulness is debatable.

(2) Visual function in aniridia

Ocular complications of aniridia include iris hypoplasia, macular hypoplasia, keratopathy, cataract, microphthalmia, nystagmus, and glaucoma. However, it is unclear to what extent each ocular complication affects visual function.

(3) Treatment options for keratopathy (corneal stromal opacity)

Keratopathy, one ocular complication of aniridia, includes corneal stromal opacity and limbal stem cell deficiency. Penetrating keratoplasty may be performed as treatment for corneal stromal opacity. However, the optimum treatment among the various treatment options has not been determined.

(4) Treatment options for keratopathy (limbal stem cell deficiency)

Keratopathy, one ocular complication of aniridia, includes corneal stromal opacity and limbal stem cell deficiency. Limbal transplantation and cultivated epithelial transplantation may be performed as treatments for limbal stem cell deficiency. However, the optimum treatment has not been determined.

(5) Treatment options for cataract

In aniridia, cataract surgery is often more difficult than usual due to corneal opacity and a shallow anterior chamber. Keratopathy may also progress as a result of surgical invasion. Therefore, it is necessary to clarify whether cataract surgery or follow-up should be selected.

(6) Treatment options for glaucoma

The treatment of glaucoma, one ocular complication of aniridia, includes the use of eye drops, oral drugs, and surgical treatment. Surgical treatment is performed when there is a lack of response to eye drops and oral drugs. Surgical treatment includes trabeculotomy, trabeculectomy, and implant surgery and is selected according to the patient’s age, residual visual field, intraocular pressure, and background factors. Each type of surgery has its own adverse effects and complications, and it is unclear which treatment is most appropriate. Therefore, clarification is required.

(7) Treatment options for low vision

Patients with aniridia often complain of low vision. The appropriate type of low vision care has not been determined.

(8) Treatment options for photophobia

Patients with aniridia often complain of photophobia. The appropriate type of care for photophobia has not been determined.

(9) Extra-ocular complications in aniridia

Aniridia may be associated with extra-ocular complications such as callosal agenesis, epilepsy, higher brain dysfunction, anosmia, glucose intolerance, and Wilms’ tumor; however, the frequency is unknown.

7. Scope of the guideline

Patients diagnosed with aniridia

8. List of clinical questions

CQ1: Is keratoplasty recommended for corneal stromal opacity in aniridia?

CQ2: Is surgical treatment recommended for limbal stem cell deficiency in aniridia?

CQ3: Is surgical treatment recommended for cataract in aniridia?

CQ4: What treatment options are appropriate for high intraocular pressure and glaucoma in aniridia?

CQ5: What is the recommended form of low vision rehabilitation in aniridia?

CQ6: What is the recommended treatment for photophobia in aniridia?

#### V. Information regarding the systematic review

1. Search schedule

Literature search: November–December 2018

Literature screening: December 2018–June 2019

Evaluation of overall evidence and summary: July–September 2019

2. Search for evidence

(1) Types of evidence

The search included existing clinical practice guidelines, systematic review (SR)/meta-analysis articles, and individual research articles, in order of priority. Randomized controlled trials (RCTs), non-randomized controlled trials, observational studies, and case series were included as individual research articles.

(2) Databases

The search was conducted in Medline (OvidSP), The Cochrane Library, and Ichushi-Web. Articles that were not stored in these databases were also included if cited.

(3) Basic search strategy

To fully cover existing guidelines and SR/meta-analysis articles, etc., and to prevent articles from being omitted from the search, a general search was conducted initially, followed by an individual search for each CQ. All databases were searched from their inception unless otherwise specified. The literature search included articles published in English and Japanese.

3. Inclusion and exclusion criteria for literature

Existing guidelines and SR articles that met the inclusion criteria were prioritized. If there were no existing guidelines or SR articles that met the criteria, an SR was conducted independently for individual research articles (*de novo* SR). In the *de novo* SR, priority was given to RCTs that met the inclusion criteria. If no RCT met the criteria, observational studies were included. Depending on the CQ, case series and case reports were also included.

4. Evaluation method and summary of evidence

The overall strength of the evidence was assessed via the method described in the “Minds Guide for Developing Clinical Practice Guidelines 2017.” The integration of the overall evidence was presented qualitatively and, where appropriate, quantitatively.

#### VI. Preparation of the recommendations, finalization, and release

1. Basic policy for the preparation of recommendations

Recommendation decisions were based on the deliberations of the guideline development group. If no consensus was reached, a vote was conducted. In addition to the “strength of evidence” and “balance between benefits and harms,” the “diversity of patient values” and “economic perspective” were also taken into consideration for determining recommendations and their strength.

2. Finalization

An external review was conducted. Public comments were solicited, and the results are reflected in the final version.

3. Specific method of external review

The external review committee members submitted comments individually. The guidelines’ development group discussed whether the clinical practice guidelines needed to be modified for each comment and decided on the action to be taken. Similarly, the guidelines’ development group discussed the need to modify the clinical practice guidelines for each public comment and decided on the action to be taken.

4. Plan for release

After completing the external review and processing the public comments, the guidelines’ supervisory committee decided on the final release. The method of release was decided after discussions between the guidelines’ development group and guidelines’ supervisory committee.

(Yoshinori Oie)

## Chapter 2

### Recommendations

#### − Diagnosis −

**Table:** 

**Background Question 1.**	
**Is genetic testing useful for diagnosing aniridia?**	
(Akira Murakami, Motokazu Tsujikawa, Toshimitsu Kasuga, Kanji Hori).	

A literature search was conducted focusing on genetic research, genetic analysis, and genetic testing of patients with aniridia. Articles published in languages other than English or Japanese were excluded. There are 2 types of aniridia: one with no abnormalities other than in the eye and one that appears as a symptom of Wilms’ tumor-aniridia-genital anomalies-retardation (WAGR) syndrome. Because both types are related to the *PAX6* gene, we also searched for WAGR syndrome. The diagnosis of Wilms’ tumor and the risk of developing extra-ocular disease were not examined. The genetic test for aniridia and its usefulness are described below, based on the results of the SR, which found case studies and review articles.

1. Genetic analysis of aniridia

Many articles related to genetic testing for aniridia describe one or more of the following approaches: *PAX6* sequencing and multiplex ligation-dependent probe amplification (MLPA) in and around the entire *PAX6* gene, immunostaining, fluorescent in situ hybridization, and chromosomal microarray (CMA) to detect chromosomal aberrations in the 11p13 region.

2. *PAX6*-related aniridia

*PAX6*-related aniridia is a disease that can cause panocular morphological abnormalities. If there are ocular findings in aniridia but no abnormalities other than the ocular findings (often referred to as isolated aniridia, which does not imply a genetically sporadic case), genetic diagnosis is confirmed once pathological mutations in *PAX6* are shown, i.e., a heterozygous one-base substitution (stop codon mutation, missense mutation, or splice mutation), one-base deletion, one-base insertion, partial gene deletion, or total deletion is observed. In addition, if other clinical findings due to WAGR syndrome are observed, the diagnosis of aniridia is confirmed if deficiencies in the *PAX6* gene and *WT1* gene are confirmed [13–55].

3. Genetic testing for aniridia

Various degrees of iris hypoplasia, nystagmus, macular hypoplasia, cataract complications, small cornea, and severe refractive error are known; however, at present, definitive diagnosis based on clinical findings alone is considered difficult. In some patients with aniridia, the macula is well formed with only a slight change in the iris, and the eyesight is good. Therefore, in patients with aniridia it is difficult to accurately estimate the ratio of each mutation to no mutation in the *PAX6* gene. According to reports of analyses with Sanger sequencing or next-generation sequencing, some mutations were found in nearly 85% of patients with isolated aniridia [20, 28, 39, 40, 44]. Of these, known mutations or mutations that clearly affect *PAX6* function, such as nonsense mutations, have high diagnostic value. The interpretation of new mutations and missense mutations may require further accumulation of genetic information. In addition, defects in the *PAX6* gene or adjacent regions have been detected in nearly 15% of cases by performing MLPA or CMA. The detection sensitivities of MLPA and CMA are unknown; however, any abnormality detected has great diagnostic significance [44]. Aniridia without involvement of the *PAX6* gene has also been reported, although it is rare [55, 56].

4. Genetic testing of WAGR syndrome

Nearly one-third of all cases of aniridia have been shown to be part of the WAGR syndrome. Genetic testing is recommended if there is no family history of aniridia or if clinical findings suggest WAGR syndrome [20, 28, 39, 40, 44]. If a gene defect is detected in *PAX6* and the neighboring *WT1*, frequent routine examinations can be performed during the ages at which the risk of developing Wilms’ tumor is high. This approach also facilitates follow-up of possible developmental delays. On the other hand, if no corresponding chromosomal structural abnormality or gene deletion is confirmed and a pathological mutation is detected via *PAX6* sequencing, it can be assumed that there is no chance that WAGR syndrome will develop. Chromosomal tests have traditionally been performed as cytogenetic tests [36]; however, CMA, which detects copy number alterations in DNA, may be useful as an alternative test.

5. Precautions for genetic testing

In a system in which not only DNA sequencing but also the detection of structural abnormalities of the genome can be used as clinical tests, appropriate genetic counseling and genetic testing for *PAX6* and its neighboring upstream genes, performed by experts, are considered useful in the treatment of aniridia and WAGR syndrome.

In GeneReviews^®^, the scenario in which genetic testing is performed for aniridia is described as an expert opinion [44]. In Japan, most medical treatment is carried out through the social insurance system; thus, it is necessary to consider which genetic tests should be recommended.

#### − Ocular complications −

**Table:** 

**Background Question 2.**	
**Which factors are the most important for visual function in patients with aniridia?**	
(Yoshinori Oie, Ryo Kawasaki)	

Ocular complications of aniridia include iris hypoplasia, macular hypoplasia, keratopathy, cataract, microphthalmia, nystagmus, and glaucoma. However, it is unclear to what extent each ocular complication affects visual function. Therefore, an SR was performed to assess the outcomes of decreased quality of life (QOL) and deterioration of long-term visual acuity prognosis.

1. Decreased QOL

The following is a summary of the results of studies that evaluated visual acuity as an index of declining QOL. In patients with aniridia, multiple ocular complications often occur simultaneously, and keratopathy, cataract, and glaucoma are known to progress with age. Hence, there are some limitations in conducting an independent analysis of the relationship between each ocular complication and visual acuity.

(1) Glaucoma

Studies show that glaucoma is associated with decreased visual acuity, and several of these studies are described here. In a case series study of 306 patients, none of the patients with glaucoma had a better visual acuity than 20/60 [57]. In another case series study, 10 out of 33 patients (30%) had glaucoma as a complication; it was the main cause of visual impairment, and 2 patients (6%) were blind [58]. Uncontrolled elevated intraocular pressure is associated with progressive visual impairment [59]. A significant correlation between final visual acuity and elevated intraocular pressure was found in 60 patients who underwent long-term follow-up [60]. Patients who needed surgery for glaucoma had poor visual acuity [61]. From these data, it is highly possible that glaucoma is related to a deterioration in visual function.

(2) Macular hypoplasia

Macular hypoplasia has been studied as discussed below. A decreased macular reflex is reported to be associated with decreased visual acuity [62]. Severe macular hypoplasia was also associated with low vision, as observed on spectral-domain optical coherence tomography (SD-OCT) [63], and with color blindness [64]. However, in patients with macular hypoplasia, a wide range of visual acuities are reported, ranging from 0.1 to 0.7 [63, 65, 66]. Hypertrophy of the outer layer of the retina, as observed on OCT, is associated with good visual acuity and is useful for predicting visual acuity prognosis [67]. In summary, macular hypoplasia may be related to a decrease in visual acuity; however, the extent of the decrease differs among patients.

(3) Nystagmus

A case series study of 60 patients found a significant correlation between the final visual acuity and nystagmus [60].

(4) Keratopathy

In keratopathy, visual acuity was reported to deteriorate when corneal opacity affected the pupillary area [68], and corneal opacity played a role in progressive visual impairment in a case series of 31 patients [59]. Congenital central corneal opacity (COO) and aniridia-associated keratopathy (AAK) are reported in 138 patients with keratopathy, and patients with COO are reported to have significantly worse visual acuity than patients with AAK. However, patients with COO were also found to have a higher rate of glaucoma complications than those with AAK, and it is unclear whether this was an effect of keratopathy alone [69]. Visual acuity is also poor in patients requiring surgical treatment for corneal opacity [61]. Based on the above, some patients are considered to have impaired visual acuity due to keratopathy.

(5) Cataract

Cataract was reported to play a role in progressive visual impairment in a case accumulation study of 31 patients [59].

(6) Iris hypoplasia

Patients with familial aniridia with an almost normal iris had good visual function, whereas a large iris deficiency was not necessarily related to visual impairment [70]. Another study found that iris deficiency was not the cause of visual impairment in patients with aniridia in a large pedigree [62]. Another study found that some patients had less iris hypoplasia, less nystagmus, and better visual acuity [71]. On the other hand, some patients were reported to show declines in visual function even in the presence of mild iris hypoplasia [72]. Considering all the above, there appear to be individual differences among patients, although visual acuity may be good in patients with mild iris hypoplasia.

(7) Microphthalmos

No reports were found on the relationship between microphthalmos and visual acuity.

2. Worsening of long-term visual acuity outcomes

Among ocular complications, keratopathy and cataract can be treated, leading to improvements in visual function. Refer to CQ2 to CQ4 for the benefits and harms of keratoplasty and cataract surgery. Because the progression of glaucoma is irreversible, it may play a role in the worsening of long-term visual acuity outcomes.

Thus, glaucoma, macular hypoplasia, nystagmus, keratopathy, cataract, and iris hypoplasia are important ocular complications that determine the visual function of patients with aniridia. Impairment of the visual field and visual acuity due to glaucoma are irreversible, and intraocular pressure management is considered important in the follow-up of patients with aniridia. The ocular complications of keratopathy and cataract can also be treated with intervention, and, based on the recommendations in CQ2 to CQ4, medical treatment is recommended.

Finally, no reports of meta-analyses of the relationship between these ocular complications and visual acuity were found, and there is currently no clear answer to the BQ. In addition to conducting a meta-analysis, the status of iris diseases in Japan needs to be analyzed.

**Table:** 

**Clinical Question 1.**	
**Is keratoplasty recommended for corneal stromal opacity in aniridia?**	
(Jun Shimazaki, Daisuke Tomida, Shimpei Komoto)	

**Table:** 

**Presentation of recommendations**	

It is weakly recommended not to perform keratoplasty for corneal stromal opacity in aniridia. The improvement in visual function resulting from a corneal transplant is limited because of the comorbidities of aniridia. In addition, the long-term prognosis in terms of visual acuity is often poor because of glaucoma and graft dysfunction over time.

**Table:** 

**Strength of recommendation**	

Weak recommendation: not to implement

**Table:** 

**Strength of evidence for the Clinical Question**	

B (Moderate)

**Table:** 

**Development process of the recommendation**	

A review was conducted on studies that investigated penetrating keratoplasty, lamellar keratoplasty, and keratoprosthesis (type I Boston keratoprosthesis [Boston KPro]). All of the papers selected were case series or retrospective cohort studies, and some were combined with limbal transplantation [73–80]. As outcomes, improved visual function and corneal transparency were the most common, and adverse events related to keratoplasty were also considered an important evaluation parameter.

Overall, penetrating keratoplasty and lamellar corneal transplants for aniridic keratopathy provide short-term improvements in visual acuity; however, a high rate of recurrence of keratopathy has been observed. In addition, the incidence of complications or adverse events such as glaucoma, infectious keratitis, and retinal detachment is high. Visual function may be maintained for a longer period of time using a limbal transplant or Boston KPro. The prognosis of keratoplasty is poor, and it is weakly recommended that one does not perform a corneal transplant. However, for patients with severe corneal opacity, keratoplasty may be performed after carefully considering the balance between benefits and risks.

**Table:** 

**Summary of SR report**	

Corneal transplants for corneal stromal opacity in aniridia include penetrating keratoplasty, lamellar keratoplasty, and Boston KPro; however, to date, no reports have compared these approaches. The following studies were included: a case series of penetrating keratoplasty alone [73], a case series of penetrating keratoplasty performed after limbal transplantation [74], a retrospective cohort study of limbal transplantation and penetrating keratoplasty versus non-transplantation [77], a retrospective cohort study on interventions including penetrating keratoplasty and lamellar keratoplasty [75], and four case series on Boston KPro [76, 78–80].

Based on these studies, an SR was performed to investigate visual acuity improvement, corneal transparency, and adverse events. No reports of high-evidence studies, such as RCTs and meta-analyses, were found, probably because aniridia is a rare disease.

1. Improvement of visual acuity

The following studies were perused to elucidate the improvement in vision: a case series of penetrating keratoplasty alone [73], a case series of penetrating keratoplasty performed following limbal transplantation [74], a retrospective cohort study of limbal transplantation and penetrating keratoplasty versus non-transplantation [77g keratoplasty and lamellar keratoplasty [75], and four case series on Boston KPro [76, 78–80]. The studies used various definitions of visual acuity improvement, and most had no clear criteria and involved comparisons of findings before and after surgery. However, some studies that used the Snellen chart confirmed improvements at two or more stages [80] and some evaluated changes in visual acuity in the short and long term [75, 77].

Among the three studies that investigated penetrating keratoplasty, Kremer et al. report that penetrating keratoplasty alone showed improvement in visual acuity in 73% of cases after a mean follow-up of 3 years. However, the postoperative visual acuity was 0.01 or less in 80% of cases. Outcomes were affected by complications such as hypoplasia of the macula and glaucoma/cataract [73]. In a report by Holland et al., in which penetrating keratoplasty was performed after limbal transplantation, visual acuity improved after surgery, with a mean follow-up of 35.7 months. No significant differences were observed between limbal transplantation alone and concomitant penetrating keratoplasty [74]. In a retrospective cohort study conducted by de la Paz et al. of limbal transplantation and penetrating keratoplasty versus non-transplantation, no significant differences were reported in visual acuity between the transplanted and non-transplanted groups after a long-term follow-up of more than 15 years [77].

In a retrospective cohort study of interventions involving penetrating keratoplasty and lamellar corneal transplants conducted by Tiller et al., the 18.5-year long-term follow-up showed significantly better postoperative best visual acuity in the transplanted group than in the non-transplanted group. However, eventually, over time, no significant differences were observed between the two groups [75].

Akpek et al. conducted a follow-up of Boston KPro with a mean duration of 17 months and found that 14/15 patients (93%) showed improvements in postoperative visual acuity. Rixen et al. conducted a follow-up with a mean duration of 18 months and found an improvement in 85.7% of patients. Finally, Hassanaly et al. followed up patients for a mean of 28.7 months and reported an improvement in 65% [76, 78, 79]. On the other hand, in a report by Shah et al., who conducted long-term follow-up with a duration of 4.5 years, improvements in visual acuity were observed in 74% of patients at 6 months; however, visual acuity gradually decreased and improvement was seen in only 43.5% of patients by the end of the study period [80].

Overall, transplantation improves visual acuity in many patients in the short term; however, the improvement is limited because of comorbidities of aniridia such as macular hypoplasia. The long-term visual acuity prognosis is poor because of the high rate of glaucoma and an increased rate of graft failure over time in non-Boston KPro transplants.

The indirectness and risk of bias were judged to be serious, and the inconsistency was judged to be small. The level of the evidence was determined as C.

2. Clear graft rate

The following studies on the clear graft rate were included: 1 case series of penetrating keratoplasty alone [73], 1 case series of penetrating keratoplasty performed after limbal transplantation [74], 1 retrospective cohort study of limbal transplantation and penetrating keratoplasty versus non-transplantation [77], 1 retrospective cohort study including penetrating keratoplasty and lamellar keratoplasty [75], and 4 case series on Boston KPro [76, 78–80].

Criteria for clear grafts are unclear, and, among others, the following were used for evaluation: rejection reaction [73], clear graft rate [74], recurrence of corneal disease [75], graft failure [77], and anatomical maintenance rate [76, 78–80]. In addition, the use of corticosteroids and immunosuppressive drugs, which are considered to be important for maintaining graft function, varied. Use ranged from systemic administration only for multiple corneal transplants [73] and systemic administration of corticosteroids/immunosuppressive drugs [74, 77, 78] to no description [75, 76, 80] and no administration [79].

Among the 3 reports included for penetrating keratoplasty, Kremer et al. report that after penetrating keratoplasty alone graft rejection was seen in 64% of patients after a mean follow-up of 3 years [73]. According to the report by Holland et al., the graft failure rate was 30% in patients who received penetrating keratoplasty after limbal transplantation, with a mean follow-up of 35.7 months. This study also demonstrates that transparency was maintained for a significantly longer period of time when immunosuppressive drugs were used [74]. A retrospective cohort study by de la Paz et al. of limbal transplantation and penetrating keratoplasty versus non-transplantation compared the median long-term survival curve over 15 years between limbal transplantation and penetrating keratoplasty. Although no significant difference was observed between the 2 groups, long-term stability was better in the limbal transplantation group (48 months vs. 24 months, p = 0.78) [77].

A retrospective cohort study by Tiller et al. that included penetrating keratoplasty and lamellar corneal transplants reports a recurrence of keratopathy to varying degrees in all patients at 18.5 years [75].

According to Akpek et al., the anatomical retention rate of Boston KPro after a mean observation period of 17 months was 100%. Similarly, Rixen et al. report a retention rate of 100% after 18 months of observation, and Hassanaly et al. report a rate of 77% after 28.7 months of observation [76, 78, 79]. Shah et al. also report a good result of 87% after a long-term follow-up of 4.5 years [80].

Overall, penetrating keratoplasty and lamellar keratoplasty without limbal transplantation are associated with a trend of poor long-term clear graft rates. When keratoplasty is combined with limbal transplantation or Boston KPro, the clear graft rate is maintained for a more extended period. Although the dose and administration were not consistent across studies, many studies found that systemic administration of corticosteroids and immunosuppressive drugs should be considered for the long-term maintenance of clear grafts.

The indirectness and risk of bias were judged to be serious, and the inconsistency was judged to be small. The level of the evidence was determined as C.

3. Adverse events

The following reports about adverse events were included: a case series of penetrating keratoplasty alone [73], a case series of penetrating keratoplasty after limbal transplantation [74], a retrospective cohort study of limbal transplantation and penetrating keratoplasty versus non-transplantation [77], a retrospective cohort study on interventions including penetrating keratoplasty and lamellar keratoplasty [75], and four case series on Boston KPro [76, 78–80]. Adverse events reported include glaucoma, vitreous hemorrhage, choroidal hemorrhage, retinal detachment, and infectious keratitis.

In both types of keratoplasty, some patients’ surgery was complicated by glaucoma, and the incidence of postoperative glaucoma was high (14.3–88%). Consequently, some patients required glaucoma surgery [73–80]. In particular, because it is difficult to manage intraocular pressure with Boston KPro, Rixen et al. report that glaucoma implant surgery was performed simultaneously with keratoplasty in 57.1% of patients [78]. Adverse events unique to Boston KPro include posterior membrane formation, junction dehiscence, tissue melting, and KPro prolapse. In particular, posterior membrane formation occurred as a complication at a high rate of 13.3–61% after Boston KPro surgery, and in some patients, yttrium aluminum garnet laser or membrane resection was required when visual acuity deteriorated [76, 78–80]. In addition, it is reported that transplants were maintained during follow-up in 77–100% of patients without causing serious complications such as endophthalmitis or tissue melting, which lead to phthisis bulbi.

The risk of bias was judged to be serious, and the indirectness and inconsistency were judged to be low. The level of the evidence was determined as C.

Based on the above, penetrating keratoplasty, lamellar keratoplasty, and Boston KPro can improve visual acuity in many patients with aniridia in the short term; however, the long-term prognosis is poor. In the long term, in addition to an increase in graft failure rates over time, a high rate of complications such as glaucoma negatively affects the maintenance of visual function. Therefore, we conclude that treatment should be selected after carefully considering intraocular pressure management, including glaucoma surgery, and that limbal transplantation, Boston KPro, and immunosuppressive drugs should be considered for long-term maintenance of clear grafts.

With a deterioration of visual acuity due to aging, cataract progression, and corneal opacity progression, surgery may be considered depending on the patient’s expectations.

**Table:** 

**Clinical Question 2.**	
**Is surgical treatment recommended for limbal stem cell deficiency in aniridia?**	
(Jun Shimazaki, Takashi Miyai, Takefumi Yamaguchi)	

**Table:** 

**Presentation of recommendations**	

It is weakly recommended that one implements surgical treatment for limbal stem cell deficiency in aniridia. Specifically, a certain rate of successful ocular surface reconstruction can be expected by performing allogeneic limbal transplantation or cultivated oral mucosal epithelial transplantation. In addition, when corneal stromal opacity is present, combination with a corneal transplant is often useful for improving visual acuity.

**Table:** 

**Strength of recommendation**	

Weak recommendation to implement

**Table:** 

**Strength of evidence for the Clinical Question**	

C (Weak)

**Table:** 

**Development process of the recommendation**	

Improvements of visual acuity and corneal transparency and adverse events are considered important outcomes in the surgical treatment of aniridia associated with limbal stem cell deficiency. The prognosis of visual acuity in aniridia is greatly influenced by comorbid macular hypoplasia and optic nerve damage caused by glaucoma as well as whether cataract surgery is performed. The most important factor in determining the success or failure of surgery is the improvement in corneal transparency. In addition to events that affect visual function, the burden on patients and systemic adverse events due to long-term immunosuppression need to be considered.

Surgical treatment reported for aniridia associated with limbal stem cell deficiency includes limbal transplantation (including combined keratoplasty) and cultivated oral mucosal epithelial cell sheet transplantation. These improve visual acuity and corneal transparency in many patients. However, immunological rejection is a major problem in the transplantation of allogeneic tissues, and adverse events and patient burden related to long-term systemic immunosuppression may also be a problem.

It should be noted that the included studies were retrospective and did not have a control group. In addition, aniridia has a wide range of severity and complications, and it is presumed that there is a large selection bias; furthermore, it is unclear in which kind of patients surgery had been indicated.

**Table:** 

**Summary of SR report**	

Various reports on the surgical treatment of aniridia with limbal stem cell deficiency summarize evidence on the effectiveness and complications of limbal transplantation alone, limbal transplantation combined with keratoplasty, cultivated oral mucosal epithelial cell sheet transplantation, and postoperative immunosuppression. Compared with reports on surgery in other conditions characterized by limbal stem cell deficiency, such as Stevens–Johnson syndrome and ocular surface chemical injury, few reports are available on surgery in aniridia alone; thus, a meta-analysis of the therapeutic effect with many patients was not possible. Therefore, case reports were included. Apart from the small number of cases reported in the literature, in contrast to other diseases associated with limbal stem cell deficiency, aniridia can be associated with amblyopia; thus, there is a high possibility of disease bias in terms of evaluation of visual acuity. There are also reports on the usefulness of therapeutic soft contact lenses and scleral contact lenses (CLs) as alternative treatments; however, these reports were omitted from these guidelines because they deviate from the purpose of this CQ.

1. Limbal transplantation

The treatment of limbal stem cell deficiency generally requires limbal transplantation alone if only limbal stem cell deficiency is present. However, whenever limbal stem cell deficiency is accompanied by corneal stromal opacity, penetrating keratoplasty or lamellar keratoplasty is required in addition to limbal transplantation [81, 82].

According to a report on 6 patients/12 eyes with limbal allograft for aniridia with limbal stem cell deficiency alone (follow-up period, 64.4 months), the mean logarithm of minimum angle of resolution visual acuity improved from 1.4 (0.1 to 2.8) preoperatively to 0.35 (0.0 to 1.0) after surgery [83]. Postoperative rejection is a major problem in limbal transplantation (the primary disease of the 6 eyes was aniridia in 2 eyes and corneal chemical injury in 4) [84]. All of the allografts in the above-mentioned study showed epithelial rejection; rejection occurred in 5 of the 6 eyes as the dose of systemic immunosuppressive drugs was reduced and in the remaining eye due to poor compliance. These rejections were treated by strengthening systemic/local immunosuppression (corticosteroids and immunosuppressive drugs). Therefore, the administration of immunosuppressive drugs is considered effective for preventing rejection (aniridia was present in 1/20 eyes [85], 3/31 eyes [86], and 1/6 eyes [87] in the included studies). Immunosuppressive drugs are also considered useful in aniridia with limbal stem cell deficiency. However, simultaneous surgery by way of penetrating keratoplasty and limbal transplantation yields a poor prognosis (this statement should be considered with caution because aniridia was present in only 1 of the 39 eyes studied) [88].

In addition to systemic adverse effects such as renal damage and abnormal glucose tolerance, the administration of immunosuppressive drugs also has local adverse effects. In a review paper on limbal transplantation for limbal stem cell deficiency, rejection and infectious keratitis occurred in 6–15% of patients [81]. Some case reports describe local development of bacterial infection and cytomegalovirus infection after surgery [89, 90], and caution is required when using immunosuppressive drugs in the long term.

2. Cultivated oral mucosal epithelial cell sheet transplantation

Although immunosuppressive drugs have systemic and local adverse effects, they are advantageous in that there is no risk of allo-rejection in autologous oral mucosal epithelial cell sheet transplantation. According to the interim results of transplantation of cultivated oral mucosal epithelial cell sheet in 17 eyes, the cornea became transparent in 76.4% of patients and visual acuity improved, ranging preoperatively from hand motion to 0.05 and ranging postoperatively from hand motion vision to 0.1 and improved in 88.2% of patients [91]. Although the degree of visual acuity improvement differed significantly from that reported in another study [83], selection bias due to the severity of aniridia was considered to be a likely cause.

**Table:** 

**Clinical Question 3**.	
**Is surgical treatment recommended for cataract in aniridia?**	
(Atsushi Shiraishi, Yasuhito Hayashi, Yumi Hashimoto, Takahiro Minami)	

**Table:** 

**Presentation of recommendations**	

Although some patients may experience an improvement in visual acuity following cataract surgery in aniridia, surgery is difficult because of the fragility associated with the capsule and the Zonule of Zinn. In addition, there is a high risk of possible worsening of postoperative glaucoma, anterior fibrosis syndrome (AFS), and bullous keratopathy. Therefore, it is recommended that one considers the risks associated with surgery and provides sufficient information to the patient in advance.

**Table:** 

**Strength of recommendation**	

Weak recommendation to implement 

**Table:** 

**Strength of evidence for the Clinical Question**	

D (Very weak)

**Table:** 

**Development process of the recommendation**	

Because there are no RCTs related to cataract surgery in patients with congenital aniridia, eight case accumulation studies [11, 57, 69, 92–96] and one case report [97] were included. Of these, three studies [69, 92, 93] summarize the results of cataract surgery in congenital aniridia and two studies [94, 96] summarize the clinical course of congenital aniridia, including a treatment course other than cataract surgery. In addition, two studies summarize the results of cataract surgery, including patients with aniridia other than congenital aniridia [11, 95] and another study reports on AFS [57]. Finally, one study reports on a patient who experienced corneal endothelial cell damage after haptics entered the angle following secondary insertion of an intraocular lens [97]. In each study, the equipment, intraocular lens, and surgical method (ultrasonic phacoemulsification and extracapsular extraction) were not standardized. Three studies applied an intraocular lens with an iris, which is not commonly used in Japan [11, 69, 93]. One study applied color CLs after surgery [92]. The severity of preoperative cataract varied among study reports [93, 94] and included mature cataracts and brown cataracts, as well as cataracts without descriptions [92]. Comorbid symptoms, such as keratopathy other than cataract, glaucoma, maculopathy, and nystagmus, and age at the time of surgery varied, and a variety of patients were included. Furthermore, because the reports were of case accumulation studies, all patients were considered to be those in whom ophthalmologists had determined that cataract surgery would be either effective or unavoidable. In addition, as the results of patients with poor outcomes would not be published, publication bias may exist, with a tendency toward reporting good results only.

**Table:** 

**Summary of SR report**	

The effectiveness of surgical treatment of cataract in aniridia was evaluated based on improved visual acuity and glare/photopsia.

Regarding the improvement of visual acuity following surgical treatment of cataract in aniridia, 4 studies report an improvement in more than 50% of patients who underwent surgery (range, 66–100%) [11, 69, 93, 94]. One study reports statistically significant improvement in visual acuity [92], whereas another study [95] reports a small improvement in corrected visual acuity of 25%; however, in that study visual acuity did not decrease in any patient, and uncorrected visual acuity improved in all 8 eyes [95]. In three studies, glare and photopsia existed before cataract surgery and improved significantly after surgery [11, 69, 92]. A study conducted by Reinhard et al. [95] demonstrates an improvement of glare in 11 out of 14 patients; however, no improvement was observed in 3 patients. Intraocular lenses with iris and color CLs are reported to be useful for improving these symptoms [11, 92, 93] as well as of a pupil due to fibrosis [92].

The following adverse events associated with cataract surgery in aniridia were evaluated: exacerbation of glaucoma, increased intraocular pressure, onset of cystic macular edema, decrease in corneal endothelial cell density, and onset of AFS.

A secondary increase in intraocular pressure was observed in 26.9–50% of patients who underwent cataract surgery [69, 93, 94, 96], and glaucoma surgery, such as trabeculectomy and ciliary photocoagulation, were necessary in 4–40% of patients [11, 69, 93, 96]. On the other hand, another study did not observe the onset or worsening of glaucoma in any patients among 17 eyes that underwent cataract surgery [92]. Reinhard et al. [95] report that among 5 eyes in which glaucoma was present before cataract surgery, 4 eyes developed poor intraocular pressure control after cataract surgery, and 2 of these eyes required surgical intervention. Four out of 14 eyes that did not have glaucoma before cataract surgery developed chronic glaucoma after cataract surgery, and 2 of these 14 eyes required intraocular pressure control by surgery. Intraocular lenses with an iris may also be more likely to worsen glaucoma because of the large size of the lens and the effect on the aqueous humor barrier [92, 93].

Reinhard et al. [95] report that 18% of patients developed cystic macular edema after cataract surgery. However, in that study, prophylactic non-steroidal anti-inflammatory drug eye drops were not prescribed. Other reports do not describe macular disease triggered by cataract surgery.

Reports on corneal endothelial cell density vary. First, two studies found a significant decrease before and after cataract surgery (before cataract surgery, 2,078 ± 265 cells/mm^2^ and after surgery, 1,896 ± 533 cells/mm^2^ [69]; before surgery, 3,280 ± 473 cells/mm^2^ and after surgery, 2,669 ± 850 cells/mm^2^) [92]. Qiu et al. [94] report that 8.7% of patients who underwent cataract surgery (2/23 eyes) experienced non-compensatory changes in the cornea. In contrast, another study [92] does not report decompensated changes in any patients. Park et al. [69] report that after cataract surgery, 50% of patients (4/8 eyes) demonstrated worsening of corneal opacity and periphery angiogenesis. In that study, 1 eye required keratoplasty. Sano et al. report the case of a patient who developed bullous keratopathy 5 years after the secondary insertion of an intraocular lens [97]. Haptics were considered to have supported the angle, because sulcus fixation of the intraocular lens tends to be deviated because of the anatomical structure. Accordingly, this surgical procedure must be considered very carefully.

AFS can occur in patients with aniridia who have undergone intraocular surgery in the past. In this condition, a proliferative membrane develops that extends to the anterior chamber, causing intraocular lens deviation, low intraocular pressure, and keratopathy. Tsai et al. [96] report that among 80 patients/155 eyes, 6 patients/7 eyes (4.5%, all women) developed AFS. Of these, 7 eyes underwent cataract surgery, 6 eyes underwent glaucoma implant surgery, and 4 eyes underwent penetrating keratoplasty or limbal stem cell transplant surgery. Surgery for AFS (penetrating keratoplasty and proliferative membrane removal, intraocular lens removal and replacement, retinal repositioning, etc.) was performed on 5 of these eyes. The proliferative membrane was replaced in two of these eyes, and the intraocular lens was then replaced and the proliferative membrane removed again, with no subsequent relapse. Although surgery for AFS improved visual acuity, in two eyes it was still lower than before the onset of AFS. Tsai et al. consider that AFS was caused by multiple surgery and intraocular devices, such as tube shunts. However, only one report of AFS with a cause other than aniridia exists, and the involvement of genetic abnormalities cannot be ruled out.

All of the above studies found an improvement in visual acuity and QOL after cataract surgery; however, all of them were case series studies. In addition, because case reports tend to display good results, the strength of the evidence is weak. No recommendation can be made at this time because adverse events were more common than with conventional cataract surgery, and the surgical procedure varied from report to report. Because preoperative comorbidities differ in each patient, we recommend that the surgical approach be considered carefully; that sufficient information be provided, including details on adverse events, before informed consent is obtained; and that the procedure should be carried out carefully.

**Table:** 

**Clinical Question 4**	
**What treatment options are appropriate for high intraocular pressure and glaucoma in aniridia?**	
(Akira Murakami, Akira Matsuda, Kenji Matsushita)	

**Table:** 

**Presentation of recommendations**	

To lower intraocular pressure, one or more of the following approaches should be used: (1) intraocular pressure-lowering therapy with drugs, such as eye drops and oral drugs, (2) angle surgery (goniotomy or trabeculotomy), (3) filtration surgery (mainly trabeculectomy), (4) glaucoma implant surgery, and (5) ciliary body coagulation.

When selecting treatment options, first consider drug therapy, such as eye drops and oral drugs, and thereafter pay attention to adverse effects. If treatment is ineffective, consider angle surgery. If angle surgery is difficult or unsuccessful, trabeculectomy or glaucoma implant surgery should be considered. In deciding which procedure to perform, it is recommended that one considers factors such as the condition of the affected eye, the surgeon’s level of experience, and whether or not the facility has been certified for glaucoma implant surgery. If these treatments are unsuccessful, ciliary body coagulation may be performed, but only if the benefits outweigh the risk of complications of poor visual prognosis, such as phthisis bulbi.

**Table:** 

**Strength of recommendation**		

Strong recommendation to implement

**Table:** 

**Strength of evidence for the Clinical Question**	

C (Weak)

**Table:** 

**Development process of the recommendation**	

A search was conducted for case reports, cohort studies, reviews, and expert opinion articles describing treatments for ocular hypertension and glaucoma in aniridia. Twenty-four English articles and 6 Japanese articles were included. The therapeutic effect and its complications were reviewed for each treatment modality.

**Table:** 

**Summary of SR report**	

Aniridia is a disease caused by developmental abnormalities of the entire eye often associated with conditions that cause visual dysfunction, such as cataract, nystagmus, macular hypoplasia, and glaucoma. Because the disease has an onset at a young age, visual impairment cannot be accurately measured, and no studies have evaluated interventions that help maintain visual acuity. The available studies discuss a decrease in intraocular pressure by treatment, adverse events (complications) associated with interventions, and visual acuity prognosis.

1. Intraocular pressure control with eye drops and oral medications

In one study, glaucoma was reported in 31 of 60 patients, and eye drops and oral therapy effectively controlled intraocular pressure in 12 of these patients [98]. Specifically, oral miotic drugs and carbonic anhydrase inhibitors (in use at the time of the study, in 1974) were used. However, in a later report (2002), miotic drugs were found to be ineffective, whereas sympathetic β-blockers, sympathomimetics, and prostaglandin-related drugs (latanoprost) were found to be effective [99] (description by experts; the level of evidence was not high). The same article also states that the use of brimonidine (a sympathetic α-stimulant) eye drops should be avoided in infants because of adverse effects such as coma (use in infants under 2 years old is contraindicated in Japan). If the limbal epithelial stem cells are exhausted and corneal epithelial damage is a concern, the use of preservative-free preparations should be considered (level of evidence: C).

2. Intraocular pressure control via angle surgery (goniotomy or trabeculotomy)

Goniotomy is reported to be effective for controlling ocular hypertension and glaucoma in aniridia [100–102]. In particular, based on a review of pathological aspects and comparison with the incidence in the natural course, Chen recommends prophylactic goniotomy before the onset of ocular hypertension/glaucoma. However, this recommendation was made without a comparative control group, and the level of the evidence was weak. Another study recommends trabeculotomy as the first-line surgery [103]]. However, there were differences in age between patients who opted for surgery other than trabeculotomy and those who underwent trabeculotomy, and trabeculotomy was performed more often in younger patients. On the other hand, another study found that trabeculotomy was ineffective in patients among whom the residual iris covered the trabecular meshwork [104]. This finding was thought to be due to the anterior deviation of the ciliary body observed in the natural course [98], and its pathophysiology was also confirmed by advanced diagnostic imaging equipment in recent years [105]. In addition, another study found that damage to the Zonule of Zinn and the absence of iris tissue affected the development of the crystalline lens at the time of trabeculotomy [106]. The pathological progress should be evaluated anatomically, and attention should be paid to the onset of complications. It is recommended that one performs angle surgery, such as goniotomy or trabeculotomy, as the first-line surgery (level of evidence: B).

3. Intraocular pressure control via ciliary body coagulation

Studies on cyclocryotherapy [107, 108] found that maintaining visual function after surgery was difficult because many patients developed phthisis bulbi and/or cataract. In addition, as an anatomical finding unique to aniridia, ciliary body hypoplasia was reported [105], and the risk of development of phthisis bulbi was considered to be higher than in healthy eyes. In fact, the incidence of phthisis bulbi was higher in pediatric patients with aniridia and high intraocular pressure than in other pediatric patients with glaucoma, regardless of the course of treatment [109]. In transcorneal cyclocoagulation with a laser (direct coagulation is possible because there is no iris) [110], ciliary body atrophy was observed; however, intraocular pressure control was exacerbated due to the formation of postoperative synechiae and shrinkage of the chamber angle tissue. In addition, transcorneal cyclocoagulation via argon laser [111] and cyclocoagulation performed simultaneously with vitreous surgery on the same eye [112] are reported to be successful and to provide successful intraocular pressure control. However, these were sporadic reports, and the level of evidence was not high. Because intraocular pressure control via ciliary destruction is unpredictable and there is a risk of phthisis bulbi, ciliary body coagulation should be regarded as a last resort applied only when other treatments are unsuccessful (level of evidence: C).

4. Intraocular pressure control via trabeculectomy

Several studies [113–115] report that intraocular pressure control was obtained via trabeculectomy. However, the level of evidence was not high because those studies only included a small number of cases or were mid- or short-term studies. On the other hand, some studies found that intraocular pressure could not be controlled via trabeculectomy [116, 117] and one-fourth of patients had postoperative phthisis bulbi [109], resulting in postoperative malignant glaucoma [118]. Other complications are also reported. In the pediatric eye, trabeculectomy generally yields a poor prognosis, and the pathological condition of the angle may affect this prognosis [119]. Accordingly, it is important to apply this procedure differently from glaucoma implant surgery [117] (level of evidence: C).

5. Intraocular pressure control via glaucoma implant surgery

The Baerveldt glaucoma implant and Ahmed glaucoma valve are available in Japan as glaucoma drainage devices. Molteno glaucoma implants, which are not approved in Japan, are sometimes used overseas [120]. In addition, in the past, materials approved for eye surgery were used in the Schocket procedure when glaucoma implant surgery was not an option [121]. There are several reports of glaucoma implant surgery performed to control intraocular pressure in aniridia [12, 117, 122–125]; however, there are also reports of complications, such as fibrosis at the valve site [126] and retinal detachment [123], both after insertion of the Ahmed glaucoma valve. Complications unique to glaucoma implant surgery, including corneal endothelium disorder and implant exposure, could occur; however, to date, no prospective report has been published. We need to wait for future reports. Because there is no iris, when inserting the tip of the tube into the crystalline lens, attention must be paid to contact not only the corneal endothelium but also the crystalline lens, and the procedure is known to be technically difficult.

Khaw [99] recommends the insertion of the tube in a tangential direction rather than in the central direction of the cornea (level of evidence: B).

**Table:** 

**Clinical Question 5.**	
**What is the recommended form of low vision rehabilitation in aniridia?**	
(Mayumi Sainohira, Sanae Asonuma)	

**Table:** 

**Presentation of recommendations**	

The basis for low vision rehabilitation is refraction correction for ametropia, which aims to improve visual function in aniridia. It is also recommended that one uses low vision devices, such as magnifiers, tinted lenses, low vision glasses, closed circuit television (CCTV) devices, and Iris Lenses.

※The Iris Lens is a contact lens (CL) with an artificial iris, which differs from a colored CL (Fig. 9 in CQ6).

**Table:** 

**Strength of recommendation**	

Strong recommendation to implement

**Table:** 

**Strength of evidence for the Clinical Question**	

C (Weak)

**Table:** 

**Development process of the recommendation**	

We conducted a systematic literature search on low vision rehabilitation intended to improve visual function in aniridia. Three reports of low vision rehabilitation focused on patients with aniridia [127–129]. Although some reports demonstrated the usefulness of telescopes and Iris Lenses, all papers included were mostly old and reported the findings of cross-sectional studies or case reports.

**Table:** 

**Summary of SR report**	

A systematic literature search on low vision rehabilitation aimed at improving visual function retrieved one cross-sectional study report [130] and one case report [127] demonstrating the usefulness of telescopic lenses and telescopes. The cross-sectional study was conducted among blind school students in the United States and reported on the effectiveness of using telescopes in 5/7 patients with aniridia [130]. The case study was conducted among Japanese infants and reported that, after using Iris Lens in infancy, the corrected visual acuity was 0.1(2/20) at the age of 7 years, which improved over time to 1.0 (20/20) with a telescope [127].

The following pieces of literature reported on the usefulness of Iris Lens: 3 case reports of aniridia [127–129] and 5 articles that included aniridia patients [44, 131–134]. The benefits of Iris Lens were improvement in visual acuity [127, 128, 131–133] and reduction of nystagmus [44, 127, 128, 133, 134]. Use of the Iris Lens from the early phase is effective for nystagmus, and can improve the fixation function and visual acuity [127, 128]. It also reduces nystagmus as assessed via electrooculography [127], achieves refraction correction by reducing aberration and increases the depth of focus [128], and improves monocular diplopia via the pinhole effect [134]. The possibility of improving photosensitivity and contrast sensitivity via colored CLs was also described [133]. However, aniridia tends to be associated with congenital limbal epithelial stem cell deficiency [135], which is reported to be a possible cause of aniridia-associated keratopathy [136]. Therefore, careful follow-up of limbal epithelial stem cell damage is necessary when using CLs [137].

No reports of RCTs have been published on preventive methods and rehabilitation aimed at improving visual function in aniridia. However, there is some evidence for the usefulness of low vision devices such as telescopes and Iris Lens.

1. Low vision rehabilitation in aniridia

(1) Visual function assessment

Accurate evaluation of visual function is required before prescribing vision devices, and in the presence of a refractive error, appropriate refraction correction is necessary. Children with aniridia often have severe refractive error, and aniridia is reported to be associated with myopia in at least 64% of patients [25, 138]. For amblyopia in childhood, pleoptics must also be used [44].

(2) Selection of low vision devices and training

**Fig. 1:**
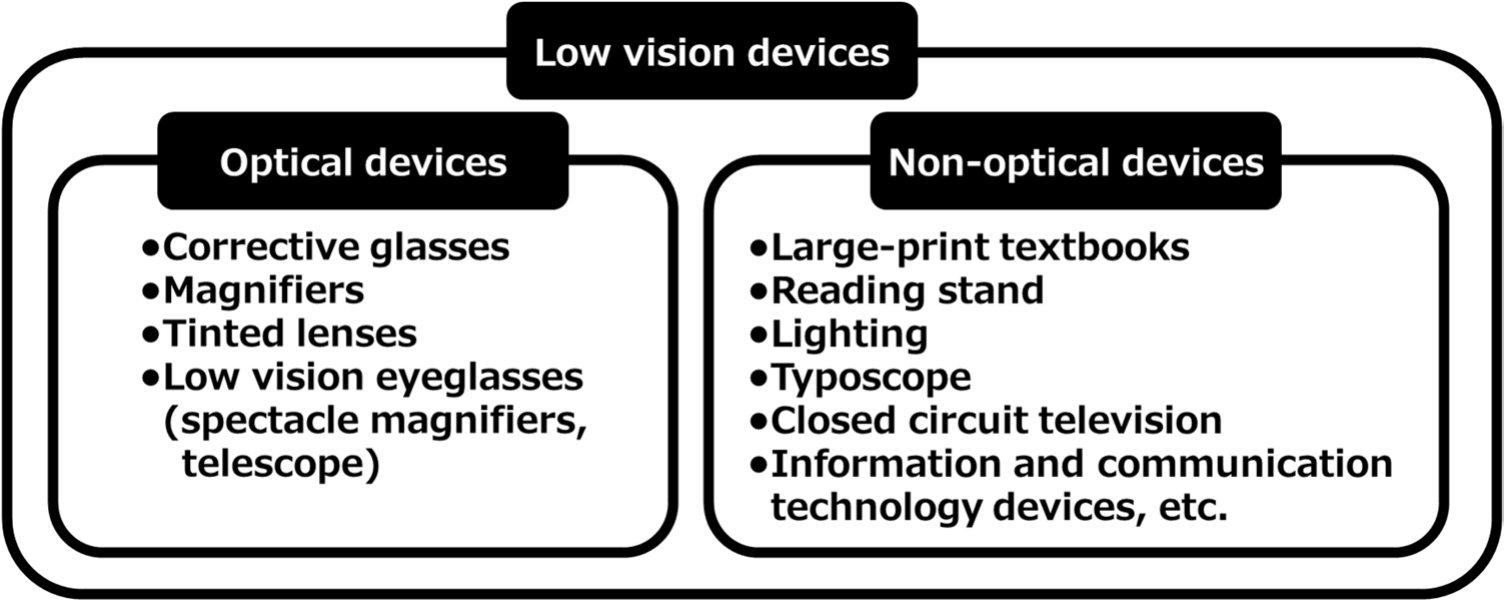
Low vision devices

**Fig. 2:**
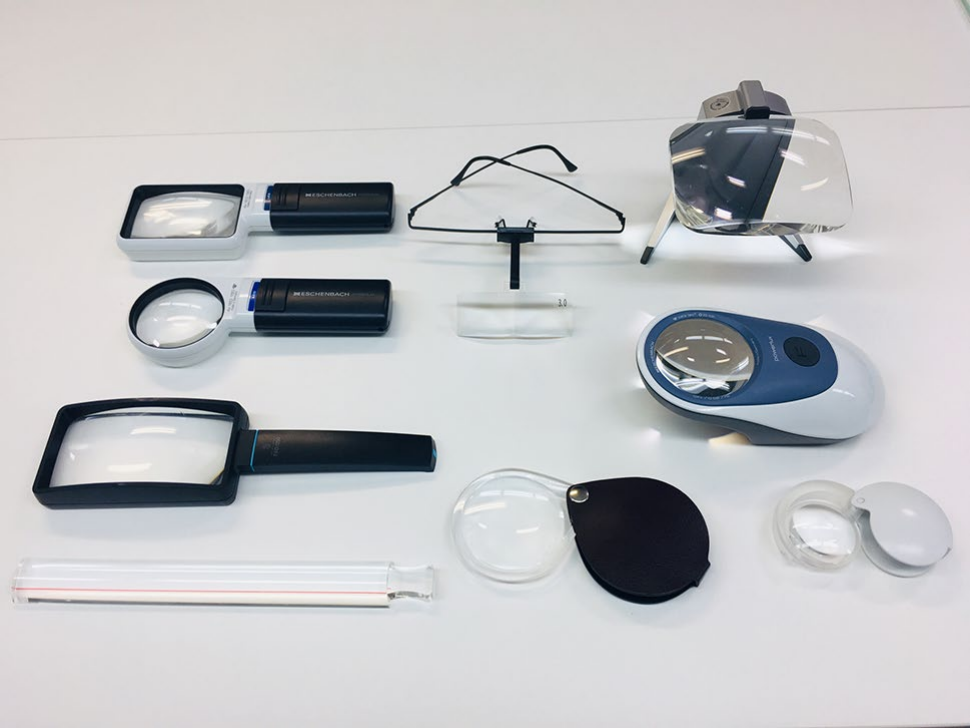
Magnifiers. Magnifiers are available in hand-held, hands-free, standing, and pocket forms. Some have LED lights

**Fig. 3:**
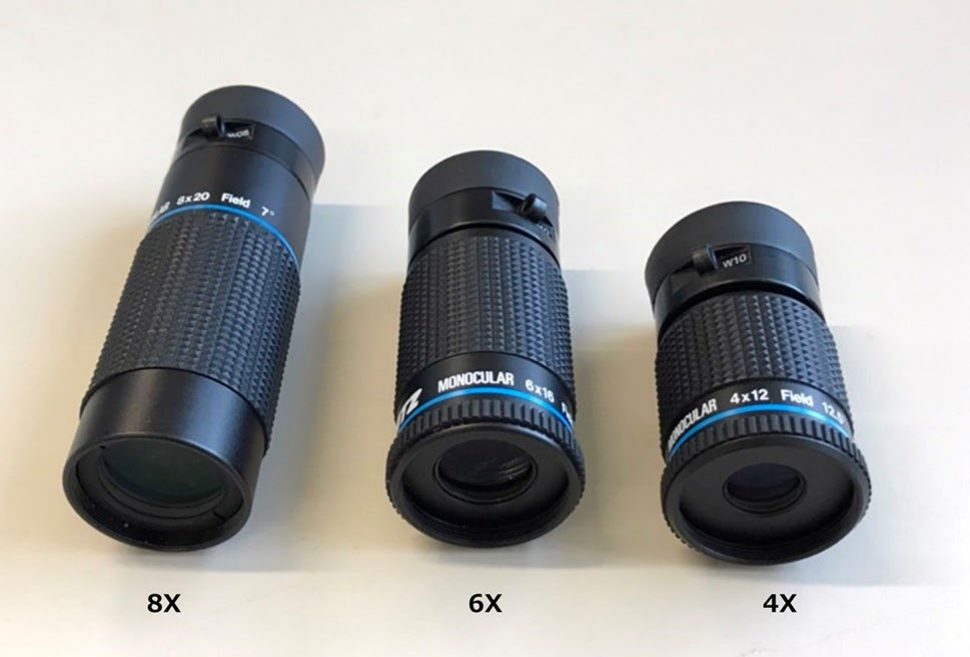
Telescopes. A telescope is the only optical device for distance viewing. It enables objects such as a television, blackboard, street signs, etc., to be seen more clearly from a distance. It allows students to copy from the blackboard because the telescope can be used with a single hand. It is provided as an orthotic device in Japan

**Fig. 4:**
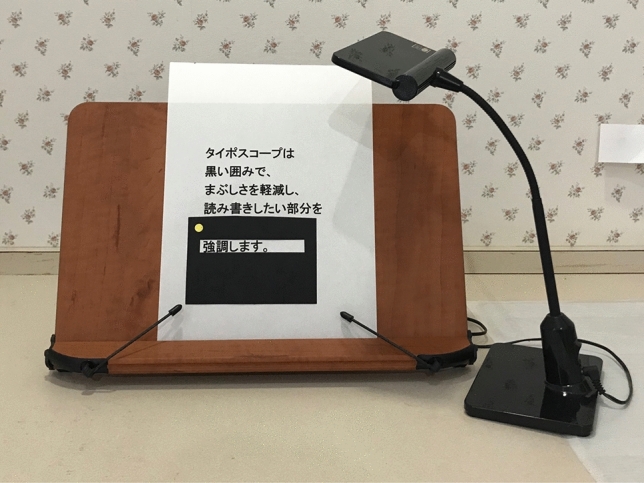
Reading stand, lighting, and typoscope. Reading stand: A reading stand can support an appropriate posture for reading and writing. It is also useful when reading Braille. Lighting: Appropriate illumination is required when reading and writing. Choose flexible lighting so that your head does not create a shadow. Typoscope: Place the part that you want to read and write in the black box. It decreases glare from the page. A patient can return to the beginning of the line by attaching the yellow circle sticker

**Fig. 5:**
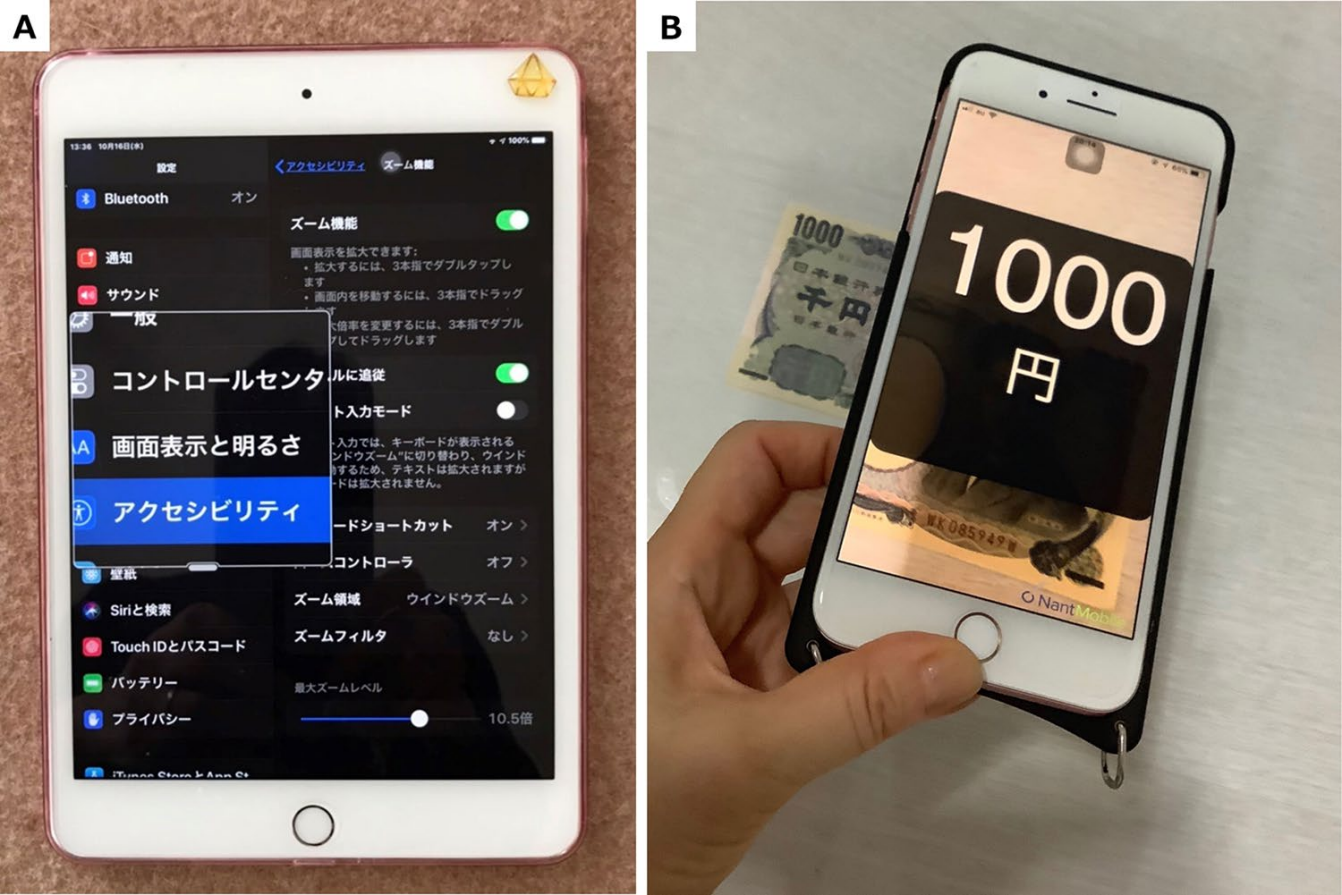
Information and communication technology devices. Mobile phones and tablet devices are equipped with accessibility functions such as brightness and zoom adjustment and voice reading as standard functions. They can also be equipped with various applications and the rear camera can be used as a portable magnifying reader. **A** Accessibility function (reverse/zoom function). **B** An application that reads out bills (money reader)

**Fig. 6:**
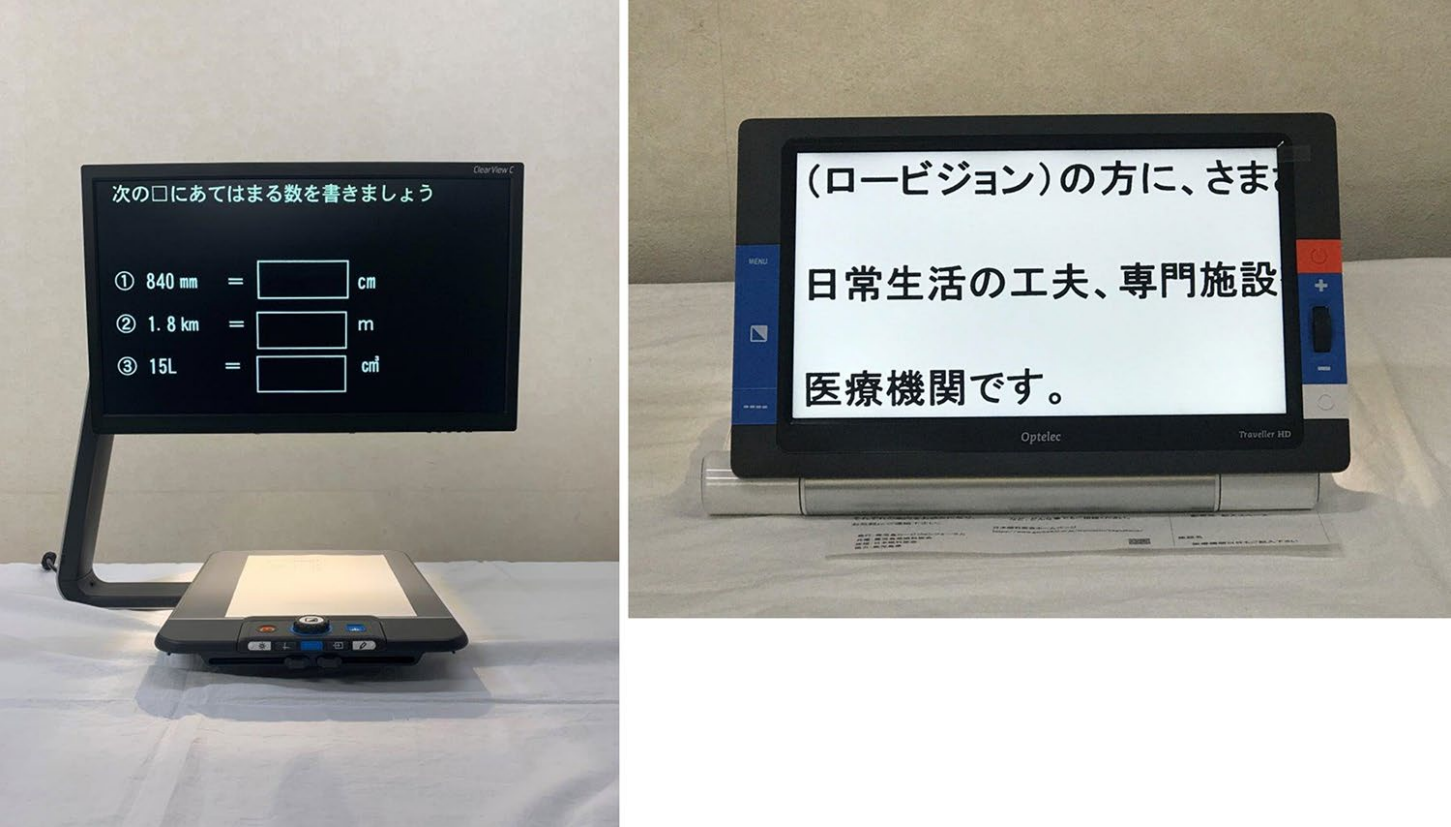
Closed circuit television. A CCTV can magnify books and textbooks to a readable size. It is provided as a tool to facilitate the daily lives of visually-impaired persons. The black-and-white inversion function (white characters on a black background) is the default and is useful for patients with photophobia

Low vision devices include optical devices (corrective glasses, magnifiers, tinted lenses, and low vision eyeglasses) and non-optical devices (such as large print textbooks, reading stands, lighting, typoscopes, CCTV devices, and information and communication technology devices) (Figs. 1, 2, 3, 4, 5, 6).

For patients of school age, it is necessary to select and use appropriate low vision aids, improve the learning environment, and provide social support according to the visual function status [44].

(3) Comprehensive rehabilitation

Comprehensive rehabilitation, by way of welfare, education, cooperation with employment-related facilities, and provision of information on social welfare systems according to life stages, is recommended [44].

Since April 2017, the Ministry of Health, Labour and Welfare in Japan has considered congenital aniridia as a designated intractable disease, and patients are eligible for medical expenses and subsidies for tinted lenses and Iris Lenses.

Orthotic devices in Japan: eyeglasses (corrective glasses, tinted lenses, and CLs [including Iris Lenses]), low vision eyeglasses (spectacle magnifiers and telescopes), white canes, and protheses.

**Table:** 

**Clinical Question 6**	
**What is the recommended treatment for photophobia in aniridia?**	
(Mayumi Sainohira, Sanae Asonuma)	

**Table:** 

**Presentation of recommendations**	

Tinted lenses and Iris Lenses are recommended as treatments for photophobia in aniridia.

*The Iris Lens is a contact lens (CL) with an artificial iris, which differs from a colored CL (Fig. 9).

**Table:** 

**Strength of recommendation**	

Strong recommendation to implement

**Table:** 

**Strength of evidence for the Clinical Question**	

C (Weak)

**Table:** 

**Development process of the recommendation**	

A systematic literature search was performed on the treatment of photophobia in aniridia. The following literature was included: three studies reporting only on aniridia patients [129, 139, 140] and seven studies that included aniridia patients as part of the study cohort [44, 127, 128, 131–134]. In these studies, Iris Lenses [127–129, 133, 134], tinted lenses [131, 132], or both were recommended as treatment for photophobia in aniridia [44, 139, 140]. These articles were mostly case reports and cross-sectional studies and were published many years ago. There were no publications of studies with high-evidence-level designs, such as RCTs and meta-analyses.

**Table:** 

**Summary of SR report**	

Based on the results of this literature search, an SR was performed on tinted lenses and Iris Lens.

1. Tinted lenses

There were 2 cross-sectional studies [139, 140] and 3 case reports [44, 127, 129] on the usefulness of tinted lenses in aniridia patients. One cross-sectional study investigated tinted lenses prescribed at the medical institution of one of the authors. Aniridia was the most common indication for which tinted lenses were prescribed in children younger than 10 years; thus, their usefulness in pediatric patients was reported [139]. Although the cross-sectional studies applied the same methodology, the number of participants was small and there was no control group.

The case reports reported an improvement of nystagmus due to photophobia, as detected by an electrooculogram, in patients who started wearing tinted lenses with a shield at the age of 3 months [127] and in patients prescribed tinted lenses because they were no longer able to wear Iris Lenses [127].

There were few reports on tinted lenses for patients with aniridia, and the level of evidence was adjudged to be weak. However, based on clinical experience, tinted lenses are preferentially recommended as a treatment for photophobia. The management of CLs may be difficult in patients with aniridia complicated by low vision and nystagmus [133], and tinted lenses are easier to handle. In addition, because there are many variations in color and design, a tinted lens can be chosen according to the individual situation. It is hoped that research on the usefulness of tinted lenses will be accumulated in the future and that their use will be scientifically verified.

2. Iris Lenses

There were only 3 case reports on the treatment of photophobia using Iris Lenses [127–129]. All the reports were of infants, including 1 in Japan [127] and 2 overseas [128, 129].

According to the case report from Japan, an Iris Lens was used in an infant at age 5 months to reduce the aggravation of nystagmus due to photophobia. As a result, the nystagmus disappeared at the age of 1 year and 2 months, and the visual acuity in both eyes was about 0.1 at the age of 7 years [127]. A case report on the use of Iris Lens in infants aged 4 to 5 months showed a reduction of nystagmus. In addition, a change in photophobia and nystagmus with and without Iris Lens was evaluated via visual inspection and video recording. Both photophobia and nystagmus decreased while the Iris Lens was used [129]. The final case report found that when infants aged 20 months used Iris Lenses, photophobia, nystagmus, and visual acuity improved [128]. All these reports stated that early postnatal use of Iris Lens may reduce nystagmus and improve visual behavior during the developmental phase.

Most of the studies [131–134] that included patients with aniridia were cross-sectional studies without a control group. Outcomes were measured in terms of the subjective usefulness (improvement of photophobia) and discomfort of wearing the CL, and the objective findings were evaluated via slit-lamp examination. The adverse effects were minor, and this method was adjudged to be extremely useful for reducing photophobia [131, 132].

On the other hand, there is a concern that Iris Lens may affect visual function. In Iris Lenses, opaque or semi-opaque tinted CLs ensure that less light reaches the retina, which may result in visual difficulties in mesopic or scotopic viewing conditions; however, there were no reports to support this concern [133].

In addition, it is reported that aniridia is often associated with congenital limbal epithelial stem cell deficiency [135] and may cause aniridia-associated keratopathy [136]. Therefore, careful follow-up of limbal epithelial stem cell damage is needed when using CLs [137].

The literature included in this report is old, and the patients’ age range was narrow. In addition, the Iris Lens and evaluation criteria used were not the same in each study. Therefore, we consider that serious indirectness was present. However, we consider that Iris Lens was consistently found to be useful for photophobia and that a certain level of evidence exists. In the future, it will be necessary to expand the age range of patients and examine adverse effects.

3. Addendum

**Fig. 7:**
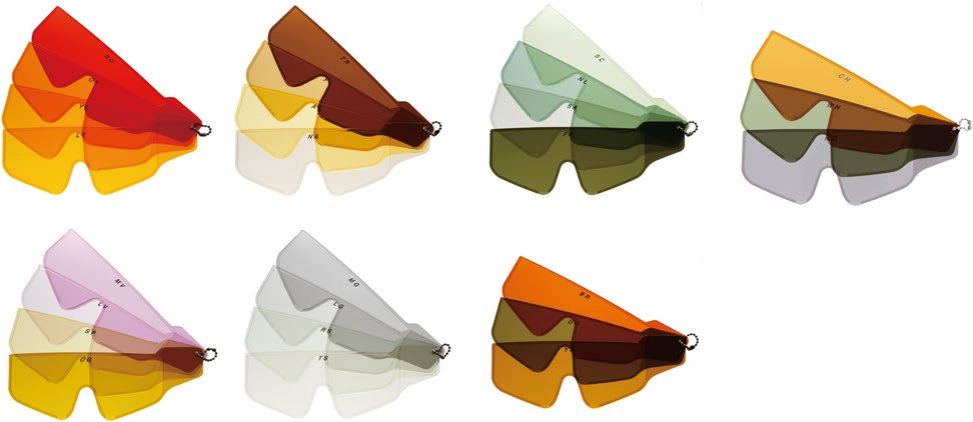
Trial tinted lenses (27 colors). Trial tinted lenses are commercially available. Patients should try them in a situation when they can actually experience photophobia and select the color that reduces the photophobia best. * Tinted lenses are defined as an aid for the purpose of reducing photophobia that partially suppresses the transmission of visible light and for which the spectral transmittance curve has been published

**Fig. 8:**
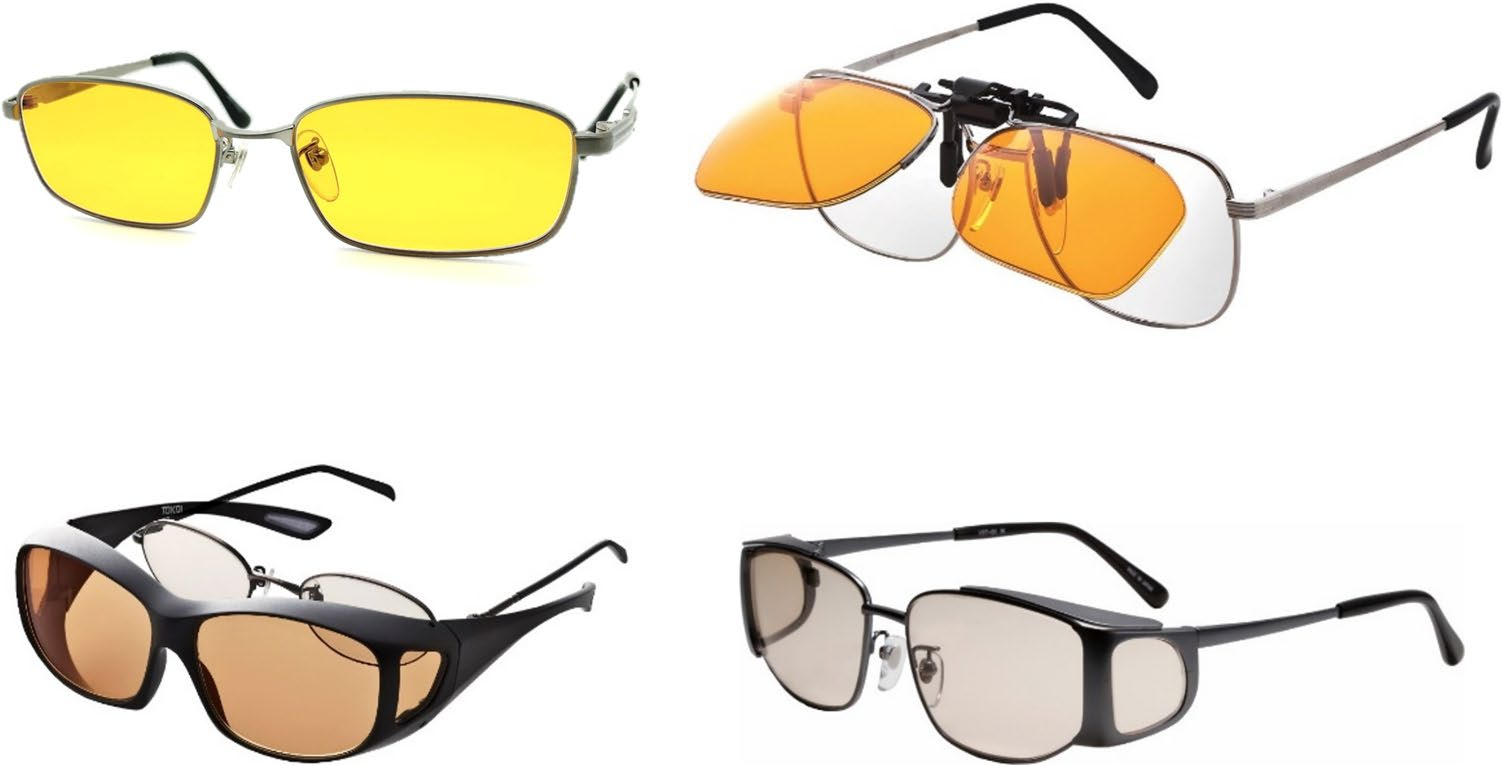
Types of tinted lenses frames. Upper left: Framed type. Tinted lens is fitted into a frame selected by the patient. Upper right: Clip-on frame. The tinted lens can be attached to a patient’s eyeglasses with a clip and can be raised or lowered as needed. One issue with this type of tinted lens is that the glasses become heavier when the lenses are attached. Lower left: Over-glass type. The tinted lenses can be worn over eyeglasses and can be used without the eyeglasses as well. Prescription lenses cannot be used. Lower right: Framed type (side shield). A light-shielding lens is fitted on the side (side shield) and the upper part of the frame is designed to prevent light from entering (top shield)

**Fig. 9:**
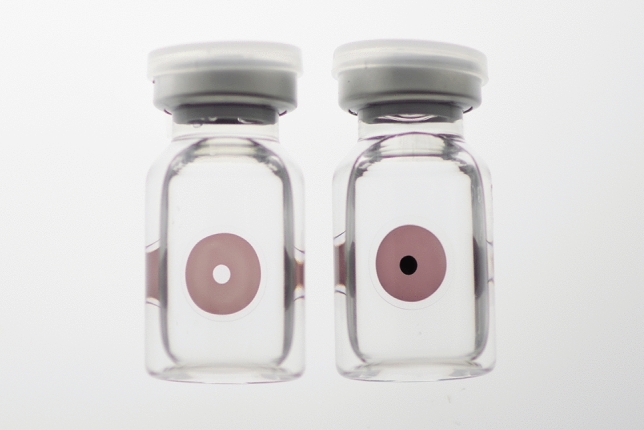
Iris Lens. The left lens is a type with a transparent pupil. The right lens has a pupil that is colored black. There are 4 iris colors for the lens, and the iris diameter, iris color, pupil diameter, and pupil color are made-to-order

Standard approaches to preventing photophobia include the use of wide-brimmed hats and umbrellas outdoors and an appropriate environment indoors with indirect lighting. As the next step, it is recommended that one wears tinted lenses or Iris Lens as needed (Figs. 7, 8, 9).

Since April 2017, the Ministry of Health, Labour and Welfare in Japan has considered congenital aniridia to be a designated intractable disease, and patients are eligible for medical expenses and subsidies for tinted lenses and Iris Lenses. Orthotic devices in Japan: eyeglasses (corrective glasses, tinted lenses, and CLs [including Iris Lens]), low vision eyeglasses (spectacle magnifiers and telescopes), white cane, and protheses.

#### − Extra-ocular complications −

**Table:** 

**Background Question 3.**	
**What is the rate of extra-ocular complications?**	
(Tomohiko Usui, Yuko Hara, Hitoha Ishii, Junko Yoshida)	

There are reports of patients with WAGR syndrome characterized by Wilms’ tumor, aniridia, genitourinary abnormalities, and mental retardation, and of patients with extra-ocular complications in aniridia. Although no studies were identified that systematically investigated the incidence of these complications, based on the results of SRs of multiple case accumulation studies, the incidences of extra-ocular complications of aniridia were as follows:

1. Wilms’ tumor

Wilms’ tumor is a malignant tumor that develops in the kidneys of children, and in patients with aniridia, the rate of Wilms’ tumor was 0–26.9% [5, 28, 49, 59, 141–146]. Friedman et al. report that routine follow-up of patients with aniridia via abdominal ultrasonography was useful for the early detection of Wilms’ tumor [142].

2. Urogenital anomalies

Studies found that 0–33.3% of patients with aniridia [5, 28, 49, 59, 141–146] had a genitourinary abnormalities. However, details were less often described. Case accumulation studies on patients with aniridia in Wilms’ tumor syndrome report findings of hypospadias, cryptorchidism, duplicate ureter, external genital abnormalities, and inguinal hernia [147, 148].

3. Mental retardation

Mental development disorder was found in 0–50% of patients with aniridia [5, 28, 49, 59, 141–146].

4. Cranial nerve abnormalities

Aniridia may be associated with complications of the cranial nerve system, such as microcephaly (9.1%) [142] and hydrocephalus (5.6%). Among cases of familial aniridia associated with *PAX6* abnormality, head MRI was performed, after which the following complication rates were reported: pineal deficiency (30%), severe cerebral dysplasia (10%), rear commissure defect (10%), and optic chiasm/corpus callosum atrophy (10%) [149].

5. Other complications

In addition, the following complications were reported: tooth dysplasia (35%), musculoskeletal abnormalities (13%), asthma (12%), depression (12%), infertility (11%, of which 6% were due to polycystic ovary syndrome), biliary disorder (8%), hypertension (8%), diabetes (7%), hyposmia (5%), and pancreatitis (1%) [5].

Upon investigating patients with WAGR syndrome, Fischbach, et al. report a variety of complications: tonsillectomy (40.7%), tympanic tube stenting enforcement (35.2%), cryptorchidism (35.2%), recurrent sinusitis (27.8%), proteinuria (25.9%), attention deficit hyperactivity disorder (22.2%), obstructive sleep apnea syndrome (20.4%), recurrent otitis media (18.5%), autism (18.5%), obesity (18.5%), occlusal abnormality (16.7%), Achilles tendon rigidity (16.7%), asthma (14.8%), spinal kyphosis/kyphosis (14.8%), abnormal tendon reflex (13.0%), recurrent pneumonia (11.1%), and obsessive-compulsive disorder (9.3%) [6].

Thus, a variety of extra-ocular complications can occur in aniridia, and many of them are related to life-long prognoses. Therefore, when examining patients with aniridia, it is important to collaborate with other departments in order to investigate extra-ocular complications.

## Abbreviations

AAK: Aniridia‒associated keratopathy
AFS: Anterior fibrosis syndrome
Boston KPro: Boston keratoprosthesis
BQ: Background question
CL: Contact lens
CMA: Chromosomal microarray
COO: Congenital central corneal opacity
CQ: Clinical question
FISH: Fluorescent in situ hybridization
MLPA: Multiplex ligation-dependent probe amplification
OCT: Optical coherence tomography
QOL: Quality of life
RCT: Randomized controlled trial
SR: Systematic review
WAGR: Wilms’ tumor‒aniridia‒genital anomalies‒retardation
